# An incremental deformation model of arterial dissection

**DOI:** 10.1007/s00285-018-1309-8

**Published:** 2018-11-19

**Authors:** Beibei Li, Steven M. Roper, Lei Wang, Xiaoyu Luo, N. A. Hill

**Affiliations:** 1 School of Mathematics and Statistics, University of Glasgow, Glasgow, UK; 2 Department of Engineering, Durham University, Durham, UK

**Keywords:** Arterial dissection, Aortic dissection, Incremental deformation, Axisymmetric tear, Residual stress, Axial pre-stretch, Holzapfel–Gasser–Ogden strain-energy, 74R99, 74B15, 74E30, 74G15, 74L15, 92C50

## Abstract

We develop a mathematical model for a small axisymmetric tear in a residually stressed and axially pre-stretched cylindrical tube. The residual stress is modelled by an opening angle when the load-free tube is sliced along a generator. This has application to the study of an aortic dissection, in which a tear develops in the wall of the artery. The artery is idealised as a single-layer thick-walled axisymmetric hyperelastic tube with collagen fibres using a Holzapfel–Gasser–Ogden strain-energy function, and the tear is treated as an incremental deformation of this tube. The lumen of the cylinder and the interior of the dissection are subject to the same constant (blood) pressure. The equilibrium equations for the incremental deformation are derived from the strain energy function. We develop numerical methods to study the opening of the tear for a range of material parameters and boundary conditions. We find that decreasing the fibre angle, decreasing the axial pre-stretch and increasing the opening angle all tend to widen the dissection, as does an incremental increase in lumen and dissection pressure.

**Electronic supplementary material:**

The online version of this article (10.1007/s00285-018-1309-8) contains supplementary material, which is available to authorized users.

## Introduction

**Fig. 1:**
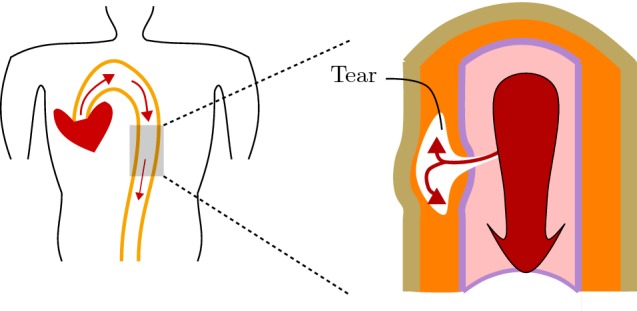
Diagrams illustrating (left) a typical creation of a dissection within the wall of the aorta, and (right) a dissection within the medial layer (orange) of the blood vessel that is connected to the lumen red, through which the blood flows, by a small medial tear. The dissection extends axially and circumferentially within the media (colour figure online)

Aortic dissection is a medical emergency and can quickly lead to death, even with optimal treatment. If the dissection tears the aorta completely open (through all three layers), massive and rapid blood loss occurs. A dissection of a large artery such as the aorta (Holzapfel 2009) is an axial and circumferential tear of the media, or between the media and adventitia, that is connected to the lumen by a small tear of the delicate intima as in Fig. 1. In addition to the original lumen for blood flow, the dissection creates a new flow channel, the ‘false’ lumen that may cause the artery to narrow or even close off entirely. If there is a tear in the overlying media and intima, blood subject to arterial pressure may then enter the false lumen and cause a more rapid and complete dissection (Benson et al. 1957). Due to the Bernoulli effect, the lateral pressure of the stagnant column of blood and clot within the dissection will exceed that in the swiftly flowing main column of blood, and Benson et al. (1957) note that a second tear in the aortic wall may follow. If the second defect breaks through the adventitia, massive haemorrhage and death usually occur. Alternatively, if the second defect breaks through the internal layer, the lateral pressure in the false lumen will drop, again in accordance with Bernoulli’s equation, and the dissection may progress.

Aortic dissections resulting in rupture have an 80% mortality rate, and 50% of patients die before they even reach the hospital. Aortic dissection is divided into acute and chronic types (Khan and Nair 2002), depending on the duration of symptoms. The aortic dissection is acute when the diagnosis is made within a fortnight after the initial onset of symptoms, and chronic thereafter. About one third of patients with aortic dissection fall into the chronic category. The most common site of initiation of aortic dissection is the ascending aorta (50%) followed by the aortic regions in the vicinity of the ligamentum arteriosum.

Only a few analytical and computational models of the arterial dissection have been introduced. Geometrically nonlinear and consistently linearised, embedded strong discontinuity models for 3D problems with an application to the dissection analysis of soft biological tissues have been described by Gasser and Holzapfel (2003). They focus on the solid mechanical and structure aspects and the geometry of the artery, and, to capture the displacement discontinuity during arterial dissection, they employ the Heaviside function and consider an enriched displacement field. This is used to investigate the propagation of a dissection in a rectangular strip from a human aortic media. Recently, Wang et al. (2015) studied the conditions for tear propagation and arrest in a 2D strip using a finite-element approach. The propagation of dissection in an infinitely-long residually-stressed cylindrical tube subject to plane strain (without axial pre-stretch), was investigated numerically by Wang et al. (2017); they found regimes in which buckling of the inner wall of the dissection occurs, as is observed clinically. In addition, Wang et al. (2018) showed that deeper dissections are more likely to propagate and that propagation occurred preferentially along mutual axes with the greatest stiffness.

In this work, we treat the dissection as an axisymmetric tear in the wall of a nonlinear hyperelastic cylindrical tube model of a large artery, whose mechanical properties are described by the strain-energy function of the arterial wall, subject to residual stress and axial stretch as *in vivo*. We ignore the small connecting tear between the lumen and the main dissection, and instead maintain both at the same constant pressure. The dissection is linearised as the incremental deformation, and the incremental nominal stress is deduced from the strain energy function of the material. The equilibrium equations, boundary conditions and jump conditions for the static dissection are derived and solved numerically. We study the model’s dependence for different parameters in the strain-energy function, and determine their effects on the opening of the tear. In our analysis, we make use of studies of the axisymmetric tear problem in an isotropic linear elastic solid by Demir et al. (1992) and Korsunsky (1995), who use a jump condition to describe the displacement discontinuity for the crack. The singular stress-displacement field resulting from the introduction of a Somigliana ring dislocation is solved by Demir et al. (1992). The Burgers vector of this dislocation has two components, one being normal to the plane of the circular ring dislocation (Volterra type) and the other being in the radial direction of the ring dislocation everywhere (Somigliana type). The analytical solution, in terms of complete elliptic integrals of the first, second and third kinds, is obtained using the Love stress function and Fourier transformation. In Korsunsky (1995), the fundamental eigenstrain solutions are derived for axisymmetric crack problems.

The strain-energy function used to describe the media of a large artery has been given by Holzapfel et al. (2000). The media is modelled as a composite, reinforced by two families of collagen fibres which are arranged in symmetrical helices. The media and adventitia respond with similar mechanical characteristics and therefore the same form of strain-energy function (but a different set of material parameters) is used for each layer. In a healthy young arterial segment (with no pathological intimal changes), the thin innermost layer of the artery is not of mechanical interest. The structure of the media gives it high strength, resilience and the ability to resist loads in both the longitudinal and circumferential directions. From the mechanical perspective (Holzapfel et al. 2000), the media is the most significant layer in a healthy artery. Dissections usually happen in the media or between the media and adventitia; we focus on a dissection in the media, and model the wall as a single layer. The artery is taken to be incompressible since it does not change volume within the physiological range of deformation.

The paper is organised as follows. We introduce the solid mechanics theory in Sect. 2, including the strain-energy function of the media of a large artery given by Holzapfel et al. (2000), and then the concepts of residual stress and axial pre-stretch in the artery, followed by the ideas of arterial dissection and incremental moduli. Next the dissection is linearised as an incremental deformation, whose traction and displacement on the tear faces and vessel boundaries are expressed as the integrals of Green’s functions weighted by the displacement discontinuity along the tear. The Cauchy stress, nominal stress, and incremental nominal stress are deduced from the strain-energy function. The Green’s functions are found numerically by Fourier transform, and the displacements along the tear faces and along the inner and outer boundaries of vessel are calculated. The numerical methods are described in Sect. 4. In Sect. 5, we present our results, in terms of the changes in the width and shape of the dissection with changes in parameter values. An increase in blood pressure is modelled as an incremental pressure difference in Sect. 6, and the consequent change in the dissection is described. Conclusions are drawn in the final section.

## Background

### Strain-energy function

The walls of the large arteries consist of three layers, the intima, media, and adventitia. According to Holzapfel et al. (2000) the intima is too thin to be of mechanical interest, and so the arterial wall is modelled as an incompressible two-layer thick-walled hyperelastic cylindrical tube with residual stress and axial pre-stretch. The two layers have similar structures, both described by an ‘HGO’ strain-energy function of the form (Holzapfel et al. 2000)

1 $$\begin{aligned} \Psi =\frac{1}{2}c\left( {\overline{I}}_1-3\right) +\frac{k_1}{2k_2} \left[ \Psi _f\left( {\overline{I}}_4\right) +\Psi _f\left( {\overline{I}}_6\right) \right] . \end{aligned}$$

The media and adventitia take different values of the material parameters $c, k_1$ and $k_2$. The parameter *c* is associated with the non-collagenous matrix of the material, and describes the isotropic part of the overall response of the tissue. The parameters $k_1$, which is an elastic modulus, and $k_2$, which is dimensionless, are associated with the anisotropic contribution of collagen to the overall response. The material parameters are independent of the geometry, opening angle and fibre angle, which are also different for the two layers. In Eq. (1),

$$\begin{aligned} {\overline{I}}_1 = \mathrm {tr}\left( {\mathbf {A}}^T{\mathbf {A}}\right) , \quad {\overline{I}}_4 = \mathrm {tr}\left( {\mathbf {M}}_+{\mathbf {A}}^T{\mathbf {A}}\right) ,\quad {\overline{I}}_6=\mathrm {tr}\left( {\mathbf {M}}_-{\mathbf {A}}^T{\mathbf {A}}\right) \end{aligned}$$

and

2 $$\begin{aligned} \Psi _f(x)=\exp \left[ k_2\left( x-1\right) ^2\right] -1. \end{aligned}$$

**Fig. 2:**
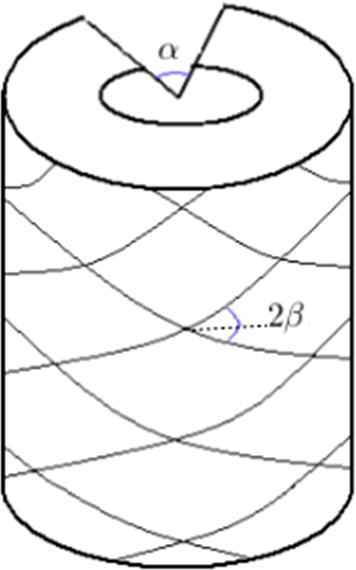
Sketch of the idealised thick-walled single-layer cylindrical model of the artery, showing the opening angle $\alpha$ and collagen fibre angle $\beta$

The tensor ${\mathbf {A}}$ is the deformation gradient, and the matrices ${\mathbf {M}}_{\pm }$ are given in cylindrical polars by

3 $$\begin{aligned} {\mathbf {M}}_{\pm }= \left[ \begin{array}{ccc} 0 &{}\quad 0 &{}\quad 0 \\ 0 &{}\quad \cos ^2\beta &{}\quad \pm \cos \beta \sin \beta \\ 0 &{}\quad \pm \cos \beta \sin \beta &{}\quad \sin ^2 \beta \end{array}\right] , \end{aligned}$$

where $2\beta$ is the angle between collagen fibres as shown in Fig. 2. In this paper we model just the medial layer, where dissections usually occur.

**Fig. 3:**
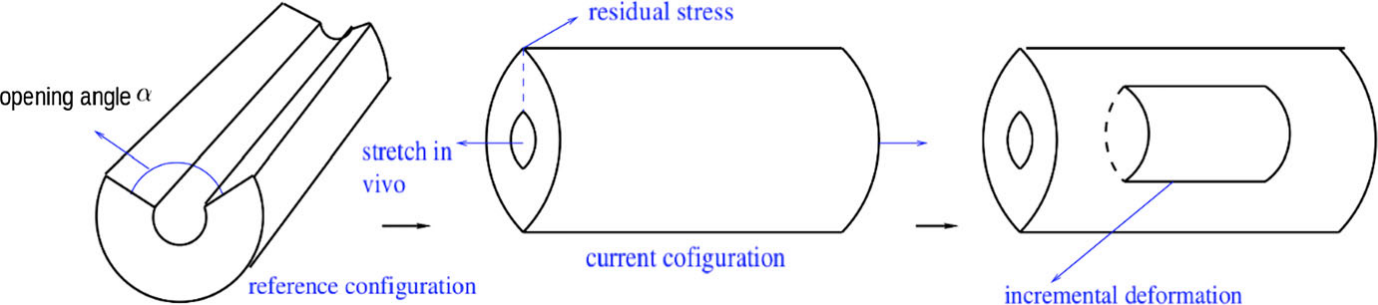
Cylindrical tube model of a large artery showing the stress-free reference configuration $\Omega _0$, the load-free current configuration $\Omega$, and the incremental deformation

### Arterial dissection and incremental moduli

The medial layer of the artery is idealized as an incompressible single-layered thick-walled cylindrical tube subject to various loads c.f. Holzapfel et al. (2000). The load-free vessel is not stress-free due to residual stress in the circumferential direction. If the arterial wall is cut along the axial direction as shown in Fig. 3, the artery opens up to some angle $\alpha$. We take the opened stress-free artery to be the reference (undeformed) configuration $\Omega _0$, and the closed artery with residual stress and axial stretch as the current (deformed) configuration $\Omega$. Cylindrical polar coordinates $(R,\Theta , Z)$ are used to describe the region $\Omega _0$: $R_\text {in}\leqslant R\leqslant R_\text {out}$, $0\leqslant \Theta \leqslant (2\pi -\alpha ),$ where $R_\text {in}$, $R_\text {out}$ and $\alpha$ are the inner and outer radii, and the opening angle, respectively. In the current configuration with polar coordinates $(r,\theta , z)$, the geometry of the deformed configuration $\Omega$ is given by $r_\text {in}\leqslant r\leqslant r_\text {out}$, $0\leqslant \theta \leqslant 2\pi$, where $r_\text {in}$ and $r_\text {out}$ are the inner and outer radii respectively. The deformation $\varvec{\chi }$, which is taken to be isochoric, is written as $\varvec{\chi }=r{{\mathbf {e}}}_r+z{{\mathbf {e}}}_z$ with reference to the basis vectors $\{{\mathbf {e}}_r, {\mathbf {e}}_{\theta }, {\mathbf {e}}_z\}$ associated with the cylindrical polar coordinates $(r, \theta , z)$, where

4 $$\begin{aligned} r=\sqrt{\frac{R^2-R_\text {in}^2}{\kappa \lambda }+r_\text {in}^2}, \quad z=\lambda Z,\quad \kappa =\frac{2\pi }{2\pi -\alpha }, \end{aligned}$$

$\lambda$ is the axial stretch, and the parameter $\kappa$ is a convenient measure of the tube opening angle in the unstressed configuration.

We treat the axisymmetric arterial dissection as an incremental deformation, and take the deformed configuration with residual stress and axial pre-stretch to be the incremental configuration shown in Fig. 3. As in Ogden (1997), when the strain energy function *W* is given, the nominal stress tensor ${\mathbf {S}}$ (which is the transpose of the first Piola–Kirchoff stress) and incremental nominal stress tensor $\delta \mathbf {S}_0$ for an incompressible material are

5 $$\begin{aligned} S_{\alpha j}= & {} \frac{\partial W}{\partial A_{j\alpha }}+qB_{j\alpha },\quad {\mathcal {A}}_{\alpha j\beta l}^1=\frac{\partial S_{\alpha j}}{\partial A_{l\beta }},\nonumber \\ {\mathcal {A}}_{0ijkl}^1= & {} A_{i\alpha }A_{\kappa \beta }{\mathcal {A}}_{\alpha j\beta l}^1, \quad \delta {S_{0ij}}={\mathcal {A}}_{0ijkl}^1\delta {A_{0lk}} -q\delta A_{0ij}+\delta q\delta _{ij}. \end{aligned}$$

(Here and elsewhere all the subscripts $i,j,k,l,\alpha$ and $\beta$ take values 1, 2 and 3.) In this case, the form of nominal stress differs from its unconstrained form, by the addition of a Lagrange multiplier *q* (the hydrostatic pressure) to enforce the constraint of incompressibility. Also in (5), ${\mathbf {B}}={\mathbf {A}}^{-T}$, $\delta {\mathbf {A}}_0$ is the incremental deformation gradient in this configuration, ${\mathcal {A}}^1_{\alpha j\beta l}$ are the elastic moduli, and ${\mathcal {A}}_{0ijkl}^1$ are the incremental moduli. Since the tissue is taken to be incompressible, $\mathrm {tr}(\delta {\mathbf {A}}_0) = 0$.

*In vivo*, the residual stress and axial pre-stretch influence the Cauchy stress and play an important role in maintaining an almost constant radial stress throughout the arterial wall. For an incompressible material, in which the deformation is given by (4), if we specify $\lambda$ and the geometry (e.g. by specifying $R_\text {in}$) then there is only one equilibrium equation (the radial component) to be satisfied, derived from the equilibrium equation

6 $$\begin{aligned} \mathrm {div}\,\varvec{\sigma }=\varvec{0}, \end{aligned}$$

and only one boundary condition is required to determine the deformation. This is

7 $$\begin{aligned} \sigma _{rr}=-P_\text {ext} \quad \text {at}\quad r=r_\text {out}, \end{aligned}$$

where $P_\text {ext}$ is the pressure on the outer boundary of the vessel. The inner pressure $P_\text {in}$ is then determined and the axial force required to produce the axial stretch $\lambda$ is also determined.

The equilibrium equation and boundary conditions for the incremental nominal stress are

8 $$\begin{aligned} \mathrm {div}\,\delta {\varvec{S}}_0 = \varvec{0}, \end{aligned}$$

and

9 $$\begin{aligned} \delta {\varvec{S}}_0^T{{\mathbf {n}}} = -{\dot{P}}{{\mathbf {n}}}+P\mathbf {\delta A}_0^T{\mathbf {n}} \end{aligned}$$

at $r = r_\text {in}$ and $r=r_\text {out}$. The pressure within the blood vessel wall is *P*. It is equal to $P_\text {ext}$ at the outer boundary and $P_\text {in}$ at the inner boundary. ${\dot{P}}$ is the incremental pressure on the boundary that specifies the change in *P*. Henceforth, for simplicity, we use $\dot{\varvec{S}}_0$ to represent $\delta {\varvec{S}}_0$ and $\dot{q}$ to refer to $\delta q$.

The deformation gradient is

10 $$\begin{aligned} {\mathbf {A}}=\left[ \begin{array}{ccc} \frac{R\left( r\right) }{\kappa r\lambda } &{}\quad 0 &{}\quad 0\\ 0 &{}\quad \kappa \frac{r}{R\left( r\right) } &{}\quad 0\\ 0 &{}\quad 0 &{}\quad \lambda \end{array} \right] =\left[ \begin{array}{ccc} a_r(r)&{}\quad 0 &{}\quad 0\\ 0 &{}\quad a_\theta (r) &{}\quad 0\\ 0 &{}\quad 0 &{}\quad a_z(r) \end{array} \right] \end{aligned}$$

where $R(r)=\sqrt{\kappa \lambda \left( r^2-r_\text {in}^2\right) +R_\text {in}^2}$. The incremental deformation is radial and axial with components *u* and *w* respectively and so the incremental deformation gradient is

11 $$\begin{aligned} \mathbf {\delta {A}_0}=\left[ \begin{array}{ccc} u,_r &{}\quad 0 &{}\quad u,_z\\ 0 &{}\quad \frac{u}{r} &{}\quad 0\\ w,_r &{}\quad 0 &{}\quad w,_z \end{array} \right] . \end{aligned}$$

As the material is incompressible, ${\text {tr}}\delta {\mathbf {A}}_0=0$. The equilibrium equation (8) is written in component form as

$$\begin{aligned} \frac{\partial {\dot{S}}_{0rr}}{\partial r}+\frac{\partial {\dot{S}}_{0zr}}{\partial z}+\frac{1}{r}({\dot{S}}_{0rr}-{\dot{S}}_{0\theta \theta })=0 \end{aligned}$$

and

12 $$\begin{aligned} \frac{\partial {\dot{S}}_{0rz}}{\partial r}+\frac{\partial {\dot{S}}_{0zz}}{\partial z}+\frac{1}{r} {\dot{S}}_{0rz}=0, \end{aligned}$$

while the boundary conditions (9) become

13 $$\begin{aligned} \left[ \begin{array}{ccc} {\dot{S}}_{0rr} &{} \quad 0 &{}\quad {\dot{S}}_{0zr} \\ 0 &{} \quad {\dot{S}}_{0\theta \theta } &{} \quad 0 \\ {\dot{S}}_{0rz} &{} \quad 0 &{}\quad {\dot{S}}_{0zz} \end{array}\right] \left[ \begin{array}{c} 1 \\ 0 \\ 0 \end{array}\right]= & {} -{\dot{P}} \left[ \begin{array}{c} 1 \\ 0 \\ 0 \end{array}\right] +P\left[ \begin{array}{ccc} u,_r &{} \quad 0 &{}\quad w,_r \\ 0 &{} \quad \frac{u}{r} &{}\quad 0 \\ u,_z &{}\quad 0 &{} \quad w,_z \end{array}\right] \left[ \begin{array}{c} 1 \\ 0 \\ 0 \end{array}\right] \end{aligned}$$

on $r=r_\text {out}$ and $r=r_\text {in}$. Note that when there is no change in the pressure boundary condition, ${\dot{P}}=0$, in which case on the outer boundary ($r=r_\text {out}$),

14 $$\begin{aligned} {\dot{S}}_{0rr}=-P_\text {ext}\left( \frac{u}{r}+w,_z\right) \quad \text {and}\quad {\dot{S}}_{0rz}=P_\text {ext}u,_z, \end{aligned}$$

and, on the inner boundary ($r=r_\text {in}$),

15 $$\begin{aligned} {\dot{S}}_{0rr}=-P_\text {in}\left( \frac{u}{r}+w,_z\right) \quad \text {and}\quad {\dot{S}}_{0rz}=P_\text {in}u,_z. \end{aligned}$$

## Static tears for an axisymmetric incompressible artery

We take the stress-free artery with opening angle $\alpha$ as the reference configuration, and the closed artery with residual stress as the current configuration. The dissection of the artery is idealised as the incremental elastic deformation on the configuration with residual stress. In the current configuration, we use $r_c$ to represent the location of the dissection. We solve a fundamental Green’s problem in which there is a point displacement discontinuity located along the tear face, c.f. Demir et al. (1992) and Korsunsky (1995). A tear is then the convolution of this fundamental solution with a density of displacement discontinuity along the tear.

For the fundamental problem, the jump conditions at $r=r_c$ are

16 $$\begin{aligned}{}[u]^+_-=\delta (z), \ [w]^+_-=0, \ [{\dot{S}}_{0rr}]^+_-=0, \ [{\dot{S}}_{0rz}]^+_-=0, \end{aligned}$$

for a jump in *u*, and

17 $$\begin{aligned}{}[u]^+_-=0, \ [w]^+_-=\delta (z), \ [{\dot{S}}_{0rr}]^+_-=0, \ [{\dot{S}}_{0rz}]^+_-=0, \end{aligned}$$

for a jump in *w*. The normal and tangential traction components $(T_{r},T_{z})$ along the dissection, are

18 $$\begin{aligned} T_{r}=\int {{\dot{S}}_{0rr}^u(z-s,r)}U(s)\,ds +\int {{\dot{S}}_{0rr}^w(z-s,r)}W(s)\,ds \end{aligned}$$

and

19 $$\begin{aligned} T_{z}=\int {{\dot{S}}_{0rz}^u(z-s,r)}U(s)\,ds+\int {{\dot{S}}_{0rz}^w(z-s,r)}W(s)\,ds, \end{aligned}$$

while the displacement components are

20 $$\begin{aligned} u= & {} \int u^u(z-s,r)U(s)ds+\int u^w(z-s,r)W(s)\,ds, \end{aligned}$$

21 $$\begin{aligned} w= & {} \int w^u(z-s,r)U(s)ds+\int w^w(z-s,r)W(s)\,ds, \end{aligned}$$

where (*U*(*s*), *W*(*s*)) is the Green’s displacement discontinuity and *s* is the arc length along the axial tear. We calculate ${{\dot{S}}}^u_{0rr}$, ${{\dot{S}}}^u_{0rz}$, $u^u$ and $w^u$ and ${{\dot{S}}}^w_{0rr}$, ${{\dot{S}}}^w_{0rz}$, $u^w$ and $w^w$ separately as follows (Li 2013). Firstly, from the strain-energy function $\Psi$, we obtain the nominal stress $\varvec{S}$ from Eq. (5) and the Cauchy stress, which is a function of the nominal stress. Next the incremental nominal stress ${\dot{\varvec{S}}}_0$ is calculated from Eq. (5). The Cauchy stress $\varvec{\sigma }$ and the incremental nominal stress ${\dot{\varvec{S}}}_0$ are functions of displacements $u^u(r,z)$, $w^u(r,z)$ or $u^w(r,z)$, $w^w(r,z)$. Writing the equilibrium equations $\mathrm {div}\,\varvec{\sigma }=0$ and $\mathrm {div}{\dot{\varvec{S}}}_0=0$ in components, we obtain partial differentiation equations with variables $u^u(r,z)$, $w^u(r,z)$ or $u^w(r,z)$, $w^w(r,z)$. We Fourier transform these PDEs into ODEs depending on the wave number *k*, with transformed variables ${\hat{u}}^u(r,k)$, ${\hat{w}}^u(r,k)$ or ${\hat{u}}^w(r,k)$, ${\hat{w}}^w(r,k)$. The ODEs are solved subject to boundary conditions and jump conditions using a collocation method. Finally, the inverse Fourier transforms are taken to obtain the solution of the original PDEs. Then ${\dot{\varvec{S}}}_0$ is given as a function of the displacement components $u^u(r,z)$, $w^u(r,z)$ or $u^w(r,z)$, $w^w(r,z)$, in terms of the incremental stress components ${\dot{S}}^u_{0rz}, {\dot{S}}^u_{0rr}$ or ${\dot{S}}^w_{0rz}, {\dot{S}}^w_{0rr}$.

Hence given the traction $({T}_{r}, {T}_{z})$ along the dissection, the displacement discontinuity (*U*(*s*), *W*(*s*)) is solved from Eqs. (18) and (19), and then the displacement (*u*, *w*) in Eqs. (20) and (21) can obtained. This method is used to calculate the displacements for the upper and lower dissection faces, and for the inner and outer boundaries.

**Fig. 4:**
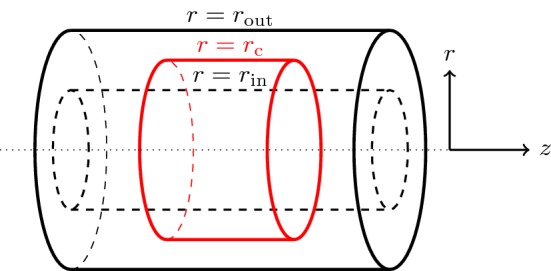
Sketch showing the location of the dissection in the current configuration before the incremental deformation is applied. The arterial wall is modelled as an axisymmetric cylinder with inner and outer radii $r_{\text {in}}$ and $r_{\text {out}}$, respectively; the dissection (shown in red) lies along the surface $r = r_c$ and $-L \le z \le L$ (colour figure online)

### Fourier transformations

We consider an axisymmetric hyperelastic cylindrical tube with inner radius $r_\text {in}$ and outer radius $r_\text {out}$. We assume the dissection, located at $r_c$, to be axisymmetric in the wall of tube, as shown in Fig. 4. The tear problem is decomposed into the normal (a jump in ‘*u*’) and tangential directions (a jump in ‘*w*’). The relevant Fourier sine and cosine transforms in the *z* direction and their inverses are defined as

$$\begin{aligned} {\hat{f}}(k,r)= & {} {\mathcal {F}}_{c,s}[f(r,z)]=\int _{0}^{\infty }f(r,z)\{\cos kz,\sin kz\}dz,\\ f(r,z)= & {} {\mathcal {F}}_{c,s}^{-1}[{\hat{f}}(k,r)] =\frac{1}{2\pi }\int _{0}^{\infty }{\hat{f}}(k,r)\{\cos {kz},\sin {kz}\}dk. \end{aligned}$$

### Jump in *w*

Using the symmetry of the domain in *z*, we express the displacements and the stresses as22and define

23 $$\begin{aligned}&{\hat{U}}^w=i{\hat{u}},\quad {\hat{W}}^w={\hat{w}},\quad {\hat{\dot{S}}}_{0rr}^w=i{\hat{\dot{S}}}_{0rr},\quad {\hat{\dot{S}}}_{0rz}^w={\hat{\dot{S}}}_{0rz}, \nonumber \\&{\hat{\dot{S}}}_{0\theta \theta }^w=i{\hat{\dot{S}}}_{0\theta \theta }, \quad {\hat{\dot{S}}}_{0zr}^w={\hat{\dot{S}}}_{0zr},\quad {\hat{\dot{S}}}_{0zz}^w=i{\hat{\dot{S}}}_{0zz}. \end{aligned}$$

The equilibrium equations (12) after Fourier transformation become

24 $$\begin{aligned}&A_1{\hat{U}}^w+B_1{({\hat{U}}^w)'}+C_1({\hat{U}}^w)''+D_1({\hat{U}}^w)''' +E_1{\hat{\dot{q}}}^w=0, \nonumber \\&A_2{\hat{U}}^w+B_2({\hat{U}}^w)'+C_2({\hat{U}}^w)''+D_2({\hat{\dot{q}}}^w)'=0. \end{aligned}$$

The transformed boundary conditions (14) and (15) are

25 $$\begin{aligned}&a_1{\hat{U}}^w + a_2{({\hat{U}}^w)'}+a_4{\hat{\dot{q}}}^w=0 \nonumber \\ \text {and}\quad&b_1{\hat{U}}^w + b_2{({\hat{U}}^w)'}+b_3({\hat{U}}^w)'' =0~\text {at}~{r=r_\text {in}}, \nonumber \\&c_5{\hat{U}}^w + c_6{({\hat{U}}^w)'}+c_8{\hat{\dot{q}}}^w=0 \nonumber \\ \text {and}\quad&d_5{\hat{U}}^w + d_6{({\hat{U}}^w)'}+d_7({\hat{U}}^w)'' =0~\text {at}~{r=r_\text {out}}, \end{aligned}$$

where $A_1$, $B_1$, $C_1$, $D_1$, $E_1$, $A_2$, $B_2$, $C_2$, $D_2$, $a_1$, $a_2$, $a_4$, $b_1$, $b_2$, $b_3$, $c_5$, $c_6$, $c_8$, $d_5$, $d_6$, $d_7$ are known functions of *r*, *k*, material parameters and deformation parameters. (See Li 2013 and Supplementary Material for further details.) The jump conditions after Fourier transformation are

26 $$\begin{aligned} \left[ {\hat{U}}^w\right] =0,~\left[ {\hat{W}}^w\right] =1,~ \left[ \hat{{\dot{S}}}_{0rr}^w\right] =\left[ \hat{{\dot{S}}}_{0rz}^w \right] =0,~\left[ {\hat{{\dot{q}}}}^w\right] =0. \end{aligned}$$

The stress components $\hat{{\dot{S}}}_{0rr}^{w}$ and $\hat{{\dot{S}}}_{0rz}^{w}$ are functions of ${\hat{U}}^w$, ${\hat{W}}^w$ and $\hat{{\dot{q}}}^w$:

27 $$\begin{aligned} \hat{{\dot{S}}}_{0rr}^{w}= & {} s_{wr1}(r){\hat{U}}^w(r)+ s_{wr2}(r)\frac{d{\hat{U}}^w(r)}{dr}+\hat{{\dot{q}}}^w, \nonumber \\ \hat{{\dot{S}}}_{0rz}^{w}= & {} s_{wz1}(r){\hat{U}}^w(r)+ s_{wz2}(r)\frac{d{\hat{U}}^w(r)}{dr}+s_{wz3}(r)\frac{d^2{\hat{U}}^w(r)}{dr^2}, \end{aligned}$$

where $s_{wr1}(r)$, $s_{wr2}(r)$, $s_{wz1}(r)$, $s_{wz2}(r)$ and $s_{wz3}(r)$ are known functions of *r*, *k*, material parameters and deformation parameters (Li 2013).

### Jump in *u*

In this case, we define

28 $$\begin{aligned}&{\hat{U}}^u={\hat{u}}\quad {\hat{W}}^u=i{\hat{w}},\quad \hat{{\dot{S}}}_{0rr}^u =\hat{{\dot{S}}}_{0rr},\quad \hat{{\dot{S}}}_{0rz}^u=i\hat{{\dot{S}}}_{0rz}, \nonumber \\&\hat{{\dot{S}}}_{0\theta \theta }^u=\hat{{\dot{S}}}_{0\theta \theta },\quad \hat{{\dot{S}}}_{0zr}^u=i\hat{{\dot{S}}}_{0zr},\quad \hat{{\dot{S}}}_{0zz}^u =\hat{{\dot{S}}}_{0zz},\quad {\hat{q}}^u={\hat{q}}. \end{aligned}$$

Applying Fourier transforms to the equilibrium equations (12) gives

29 $$\begin{aligned}&A_1{\hat{U}}^u+B_1({\hat{U}}^u)'+C_1({\hat{U}}^u)''+D_1({\hat{U}}^u)''' +E_1{\hat{\dot{q}}}^u=0, \nonumber \\&A_2{\hat{U}}^u+B_2({\hat{U}}^u)'+C_2({\hat{U}}^u)''+D_2({\hat{\dot{q}}}^u)'=0. \end{aligned}$$

The boundary conditions (14) and (15) after Fourier transformation are

30 $$\begin{aligned} a_1{\hat{U}}^u+a_2({\hat{U}}^u)'+a_4{\hat{\dot{q}}}^u=0,&\quad b_1{\hat{U}}^u+b_2({\hat{U}}^u)'+b_3({\hat{U}}^u)''=0\quad \text {at}\quad {r=r_\text {in}}, \nonumber \\ c_5{\hat{U}}^u+c_6({\hat{U}}^u)'+c_8{\hat{\dot{q}}}^u=0,&\quad d_5{\hat{U}}^u+d_6({\hat{U}}^u)'+d_7({\hat{U}}^u)''=0\quad \text {at} \quad {r=r_\text {out}}, \end{aligned}$$

where $A_1$, $B_1$, $C_1$, $D_1$, $E_1$, $A_2$, $B_2$, $C_2$, $D_2$, $a_1$, $a_2$, $a_4$, $b_1$, $b_2$, $b_3$, $c_5$, $c_6$, $c_8$, $d_5$, $d_6$ and $d_7$ are known functions of *r*, *k*, the material parameters and deformation parameters (see Li 2013 and Supplementary Material). The Fourier-transformed jump conditions yield

31 $$\begin{aligned} \left[ {\hat{U}}^u\right] =1,~\left[ {\hat{W}}^u\right] =0,~ \left[ \hat{{\dot{S}}}_{0rr}^u\right] =\left[ \hat{{\dot{S}}}_{0rz}^u\right] =0. \end{aligned}$$

The stress components $\hat{{\dot{S}}}_{0rr}^{u}$ and $\hat{{\dot{S}}}_{0rz}^{u}$, which are functions of ${\hat{U}}^u$, ${\hat{W}}^u$ and $\hat{{\dot{q}}}^u$, are given by

32 $$\begin{aligned} \hat{{\dot{S}}}_{0rr}^{u}= & {} s_{ur1}(r){\hat{U}}^u(r)+s_{ur2}(r) \frac{d{\hat{U}}^u(r)}{dr}-\hat{{\dot{q}}}^u, \nonumber \\ \hat{{\dot{S}}}_{0rz}^{u}= & {} s_{uz1}(r){\hat{U}}^u(r)+ s_{uz2}(r)\frac{d{\hat{U}}^u(r)}{dr}+s_{uz3}(r)\frac{d^2{\hat{U}}^u(r)}{dr^2}, \end{aligned}$$

where $s_{ur1}(r)$, $s_{ur2}(r)$, $s_{uz1}(r)$, $s_{uz2}(r)$ and $s_{uz3}(r)$ are known functions of *r*, *k*, material parameters and deformation parameters.

## Numerical solution

The quantities ${{\hat{U}}}^{w}$, ${\hat{W}}^{w}$, $\hat{{\dot{S}}}_{0rr}^{w}$, $\hat{{\dot{S}}}_{0rz}^{w}$, $\hat{{\dot{S}}}_{0rr}^{u}$, $\hat{{\dot{S}}}_{0rz}^{u}$, ${\hat{U}}^{u}$ and ${\hat{W}}^{u}$ describe the response to a point discontinuity in the radial (superscript *u*) and axial (superscript *w*) directions. These solutions can be used to formulate integral equations that relate the loading on the tear surfaces to the opening of the tear. In this section we describe a numerical method for the approximation of the integral equations that connect opening and loading. In order to achieve this, we must approximate the solutions to Eqs. (24)–(32). These equations, and their appropriate boundary conditions, depend parametrically on the wavenumber *k*; the case $k=0$ is a special case in which some of the coefficients in Eqs. (24)–(32) are zero. The procedure described below still applies to the case $k=0$ but with modified governing equations.

As illustrated in Fig. 4, we identify two regions; region 1 is $[r_\text {in} , r_c]$ and region 2 is $[r_c, r_\text {out}]$. In region 1 we define $r=r_1={r_\text {in}}+R({r_c-r_\text {in}})$, and in region 2 $r=r_2={r_\text {out}}+R({r_c-r_\text {out}})$. The range of *R* is [0, 1]. This allows us to transform all equations to be defined on the single domain $R\in [0,1]$. The boundaries (the internal boundary for region 1 and the external boundary for region 2) are both located at $R=0$, while $R=1$ represents the tear faces in *both* regions (though different faces depending on whether we are in region 1 or 2). The equations and boundary conditions (24)–(32) are written in terms of the new variable *R* and solved on the domain [0, 1] using the MatLab routine bvp4c. For a discrete set of wavenumbers *k*, we obtain approximations to ${\hat{U}}^{w}(r,k), {\hat{W}}^{w}(r,k), {\hat{U}}^{u}(r,k), {\hat{W}}^{u}(r,k)$ and $\hat{{\dot{S}}}_{0rr}^{w}(r,k)$, $\hat{{\dot{S}}}_{0rz}^{w}(r,k)$, $\hat{{\dot{S}}}_{0rr}^{u}(r,k)$, $\hat{{\dot{S}}}_{0rz}^{u}(r,k)$.

The traction and displacement, decomposed into normal and tangential components, and written in terms of the fundamental solutions, are

33 $$\begin{aligned} {T}_{r}(z)= & {} \int {{{\dot{S}}}_{0rr}^u\left( z-s,r\right) }U\left( s\right) ds + \int {{{\dot{S}}}_{0rr}^w\left( z-s,r\right) }W\left( s\right) ds, \end{aligned}$$

34 $$\begin{aligned} {T}_{z}(z)= & {} \int {{{\dot{S}}}_{0rz}^u\left( z-s,r\right) }U\left( s\right) ds + \int {{{\dot{S}}}_{0rz}^w\left( z-s,r\right) }W\left( s\right) ds, \end{aligned}$$

35 $$\begin{aligned} u= & {} \int u^u(z-s,r)U(s)\,ds + \int u^w(z-s,r)W(s)\,ds, \end{aligned}$$

36 $$\begin{aligned} w= & {} \int w^u(z-s,r)U(s)\,ds + \int w^w(z-s,r)W(s)\,ds. \end{aligned}$$

We discretise the integral Eqs. (33)–(36), assuming a piecewise constant tear opening along a dissection occupying $-L\le z\le L$. The tractions and displacements can be calculated anywhere, but we are particularly interested in the displacements on the inner and outer surfaces $r=r_\text {in}$ and $r=r_\text {out}$, and the tear faces $r=r_c$. We write the opening as a function of position along the tear surface as

37 $$\begin{aligned} \{W(s),U(s)\}=\sum _{j=1}^{2N}\{W_j,U_j\}{\mathbf {I}}_{\left( z_j-\Delta , z_j+\Delta \right) }(s), \end{aligned}$$

where ${\mathbf {I}}_A(s)$ is the indicator function $({\mathbf {I}}_A(s)=1$ if $s\in A$ and ${\mathbf {I}}_A(s)=0$ otherwise), $z_j=-L+\Delta +2j\Delta$ and $\Delta =L/2N$. The $z_j$ are the centres of the intervals upon which the opening is constant. Inserting these expressions into (33), we obtain for the second term

38 $$\begin{aligned} \int {{{\dot{S}}}_{0rr}^w\left( z-s,r\right) }W\left( s\right) ds= \sum _jW_j\int _j{{\dot{S}}}_{0rr}^w\left( z-s,r\right) \,ds, \end{aligned}$$

where the subscript *j* refers to an integral over the interval ${\left( z_j-\Delta ,z_j+\Delta \right) }$. The other terms are treated similarly; see Li (2013) and the Supplementary Material. Writing ${{\dot{S}}}_{0rr}^w$ in terms of its transform and changing the order of integration gives

$$\begin{aligned}&\int {{\dot{S}}_{0rr}^w\left( z_i-s,r\right) }W\left( s\right) ds\\&\quad =\int \left[ \frac{1}{\pi }\int _{0}^\infty {\hat{\dot{S}}}_{0rr}^w \left( k,r\right) \sin {k\left( z_i-s\right) }\,dk\right] \,W\left( s\right) \,ds\\&\quad =\frac{1}{\pi }\sum _j\int _j\left[ \int _{0}^\infty {\hat{\dot{S}}}_{0rr}^w\left( k,r\right) \sin {k\left( z_i-s\right) }\,dk\right] \,W_j\,ds\\&\quad = \frac{1}{\pi }\sum _j\int _j\left[ \int _{0}^\infty {\tilde{\dot{S}}_{0rr}^w}\sin {k\left( z_i-s\right) }\,dk\right] \,W_j\,ds\\&\qquad + \frac{1}{\pi }\sum _j\int _j\left[ \int _{0}^\infty k{\dot{S}}_{0rr}^{w1}\sin {k\left( z_i-s\right) }\,dk\right] \,W_j\,ds\\&\qquad + \frac{1}{\pi }\sum _j\int _j\left[ \int _{0}^\infty {\dot{S}}_{0rr}^{w0}\sin {k\left( z_i-s\right) }\,dk\right] \,W_j\,ds, \end{aligned}$$

where ${\hat{\dot{S}}}_{0rr}^w={\tilde{\dot{S}}_{0rr}^w}+k{\dot{S}}_{0rr}^{w1}+{\dot{S}}_{0rr}^{w0}$ and ${\dot{S}}_{0rr}^{w1}$ and ${\dot{S}}_{0rr}^{w0}$ are independent of *k*, and chosen so that ${\tilde{{\dot{S}}}_{0rr}^w}\rightarrow 0$ as $k\rightarrow \infty$ to ensure convergence of the quadrature used to evaluate the integrals involving $\tilde{\dot{S}}_{0rr}^w$.

$$\begin{aligned} \int {{\dot{S}}_{0rr}^w\left( z_i-s,r\right) }W\left( s\right) ds&=\frac{2}{\pi }\sum _jW_j\int _{0}^\infty \tilde{{\dot{S}}}_{0rr}^w\frac{\sin k\Delta }{k}\sin k(z_i-z_j)\,dk \\&\quad +\,{\dot{S}}_{0rr}^{w0}\sum _jW_j\frac{1}{\pi }\log \left| \frac{z_i-z_j +\Delta }{z_i-z_j-\Delta }\right| , \end{aligned}$$

which is of the form $\sum \nolimits _j{\dot{S}}_{0rr}^w[i,j]W_j$, where

$$\begin{aligned} {\dot{S}}_{0rr}^w[i,j]&=\frac{2}{\pi }\int _{0}^\infty {\tilde{{\dot{S}}}_{0rr}^w} \frac{\sin k\Delta }{k}\sin k(z_i-z_j)\,dk +{\dot{S}}_{0rr}^{w0}\frac{1}{\pi } \log \left| \frac{z_i-z_j+\Delta }{z_i-z_j-\Delta }\right| . \end{aligned}$$

The first term in (33) can be treated similarly and we obtain an expression of the form $\sum \nolimits _j{\dot{S}}_{0rr}^u[i,j]U_j$ where

$$\begin{aligned} {\dot{S}}_{0rr}^u[i,j]&=\frac{2}{\pi }\int _{0}^\infty {\tilde{{\dot{S}}}_{0rr}^u} \frac{\sin k\Delta }{k}\cos k(z_i-z_j)\,dk\\&\quad -\,{\dot{S}}_{0rr}^{u1}\left[ \frac{2\Delta }{\pi \left( \left( z_i-z_j\right) ^2- \Delta ^2\right) }\right] +{\dot{S}}_{0rr}^{u0}{\mathbf {I}}_{z_i\in (z_j-\Delta , z_j+\Delta )}. \end{aligned}$$

The second term in (34) gives

$$\begin{aligned} {\dot{S}}_{0rz}^w[i,j]&=\frac{2}{\pi }\int _{0}^\infty {\tilde{{\dot{S}}}_{0rz}^w} \frac{\sin k\Delta }{k}\cos k(z_i-z_j)\,dk\\&\quad -\,{\dot{S}}_{0rz}^{w1}\left[ \frac{2\Delta }{\pi \left( \left( z_i-z_j \right) ^2-\Delta ^2\right) }\right] +{\dot{S}}_{0rz}^{w0}{\mathbf {I}}_{z_i\in (z_j-\Delta , z_j+\Delta )}. \end{aligned}$$

Finally, for completeness, the first term in (34) gives

$$\begin{aligned} {\dot{S}}_{0rz}^u[i,j]&=\frac{2}{\pi }\int _{0}^\infty {\tilde{{\dot{S}}}_{0rz}^u}\frac{\sin k\Delta }{k}\sin k(z_i-z_j)\,dk +{\dot{S}}_{0rz}^{u0}\frac{1}{\pi }\log \left| \frac{z_i-z_j+\Delta }{z_i-z_j-\Delta }\right| . \end{aligned}$$

The discretised version of integral equations (33) and (34) are assembled into the linear system of equations

39 $$\begin{aligned} \left[ \begin{array}{c} {T_{r}}_i\\ {T_{z}}_i \end{array}\right] = \left[ \begin{array}{cc} {\dot{S}}_{0rr}^u[i,j]&{}\quad {\dot{S}}_{0rr}^w[i,j]\\ {\dot{S}}_{0rz}^u[i,j]&{}\quad {\dot{S}}_{0rz}^w[i,j] \end{array}\right] \left[ \begin{array}{c} U_j\\ W_j \end{array}\right] \end{aligned}$$

where $i=1,\ldots , 2N$ and $j=1,\ldots ,2N$. The entries in the $4N\times 4N$ matrix are given above. In an analogous manner, we discretise the integral equations (35) and (36) assuming the same piecewise constant separation of the tear faces. The displacements are evaluated at points along the inner and outer surfaces ($r=r_\text {in}$ and $r=r_\text {out}$). In exactly the same manner as for (33) and (34), we obtain the discretised integral equations for displacement.

The first term of (35) has the form $\sum \nolimits _j u^u[i,j]U_j$ with

$$\begin{aligned} u^u[i,j]&=\frac{2}{\pi }\int _0^\infty ({\hat{U}}^u-u^{1u}) \frac{\sin k\Delta }{k}\cos k\left( z_i-z_j\right) dk +u^{1u}{\mathbf {I}}_{\left( z_j-\Delta ,z_j+\Delta \right) }(z_i). \end{aligned}$$

The second term of (35) has the form $\sum \nolimits _j u^w[i,j]W_j$ with

$$\begin{aligned} u^w[i,j]&=\frac{2}{\pi }\int _0^\infty ({\hat{U}}^w-u^{1w}) \frac{\sin k\Delta }{k}\sin k\left( z_i-z_j\right) dk +\frac{u^{1w}}{\pi }\log \left| \frac{z_i-z_j+\Delta }{z_i-z_j-\Delta }\right| . \end{aligned}$$

The first term of (36) has the form $\sum \nolimits _j w^u[i,j]U_j$ with

$$\begin{aligned} w^u[i,j]&=\frac{2}{\pi }\int _0^\infty ({\hat{W}}^u-w^{1u}) \frac{\sin k\Delta }{k}\sin k\left( z_i-z_j\right) dk +\frac{w^{1u}}{\pi }\log \left| \frac{z_i-z_j+\Delta }{z_i-z_j-\Delta }\right| . \end{aligned}$$

Lastly, the second term of (36) has the form $\sum \nolimits _j w^w[i,j]W_j$ with

$$\begin{aligned} w^w[i,j]&=\frac{2}{\pi }\int _0^\infty ({\hat{W}}^w-w^{1w}) \frac{\sin k\Delta }{k}\cos k\left( z_i-z_j\right) dk +w^{1w}{\mathbf {I}}_{\left( z_j-\Delta ,z_j+\Delta \right) }(z_i). \end{aligned}$$

The integral equations (35) and (36) can now be written as a linear system of equations

40 $$\begin{aligned} \left[ \begin{array}{c} u_i\\ w_i \end{array}\right] = \left[ \begin{array}{cc} u^u[i,j]&{}\quad u^w[i,j]\\ w^u[i,j]&{}\quad w^w[i,j] \end{array}\right] \left[ \begin{array}{c} U_j\\ W_j \end{array}\right] . \end{aligned}$$

where $u_i$ and $w_i$ are the values of the displacements at a set of points indexed by $i=1,\ldots , M$, for example we choose points along the inner and outer surfaces. The matrix in (40) is an $M\times 4N$ matrix.

### Conditions at the tear face

In order to calculate the opening of the tear in response to loading we must solve Eq. (39) with prescribed tractions to obtain $U_j$ and $W_j$. This requires the traction conditions at the tear faces. When $U_j$ and $W_j$ have been obtained, the displacements $u_i$ and $w_i$ in (40) can be calculated. In our static tear, we use the following traction conditions at the dissection face. We imagine that the fluid inside the tear is connected with the fluid in the artery ($r\le r_\text {in}$). The pressure on the arterial wall in the absence of the tear is $-\sigma _{rr}(r_\text {in})$ and the pressure at radius where the tear is introduced is $-\sigma _{rr}(r_c)$. Therefore the traction conditions for the incremental problem at the tear faces are

$$\begin{aligned} {T_{r}}_i=\sigma _{rr}(r_\text {in})-\sigma _{rr}(r_{c}) \quad \text {and} \quad {T_{z}}_i=0. \end{aligned}$$

### Convergence of the Numerical Solution

Tables 1 and 2 demonstrate the convergence of the numerical scheme for a representative set of parameter values, namely $r_\text {in}=4\,\mathrm {mm}$, $r_c=5\,\mathrm {mm}$, $r_\text {out}=6\,\mathrm {mm}$, $R_i=3.9\,\mathrm {mm}$, $k_1=2.3632\,\mathrm {kPa}$, $k_2=0.8393$, and $c=3\,\mathrm {kPa}$, $P_\text {ext}=0\,\mathrm {kPa}$, $\beta =\pi /3$ and $\kappa = 1$. The numerical solutions were tested for convergence with $k_\text {max}$ and $N_k$, where $[0, k_\text {max}]$ is the range of wavenumbers used in the simulation and $N_k$ is the number of integration points, so that the wavenumber step size is $\Delta k = k_\text {max}/N_k$. When $k_\text {max} = 10$ and $N_k = 300$, the expected relative error is less than 0.1%, and so these values are used to generate the results below, for computational efficiency.

**Table 1:** Convergence of numerical scheme with maximum wave number ($k_\text {max}$) and number of intervals ($N_k$) in $[0, k_\text {max}]$

$k_\text {max}$	$N_k$	Maximum dissection width
10	300	0.233178
10	500	0.233256
20	1000	0.233259
30	500	0.232907
30	1000	0.233203
30	1500	0.233259

**Table 2:** Convergence of numerical scheme with $N_k$ for fixed maximum wave number $k_\text {max} = 10$

$N_k$	Maximum dissection width
500	0.233256
600	0.233270
700	0.233278
800	0.233284
900	0.233287
1000	0.233290
1200	0.233293
1500	0.233296

### Validation of the numerical scheme

**Fig. 5:**
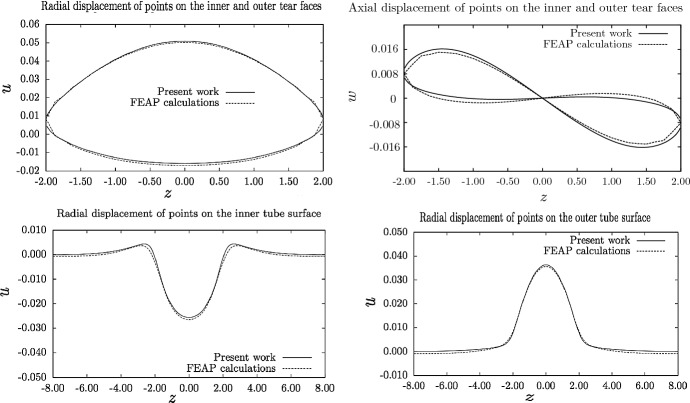
A comparison of the results of the numerical method described here, with the results from the finite-element program FEAP for a tube of finite length. All lengths are in mm. (See text for parameter values)

The numerical method described in Sect. 4 for an infinitely long tube has been validated by comparison with a finite-element calculation for the special case of a tube of fixed length, in the absence of axial pre-stretch ($\lambda = 1$) and residual stress ($\kappa = 1$), using the numerical software package FEAP (Taylor 2011). Details of the implementation of the HGO strain-energy function in FEAP are given by Wang et al. (2015). In this comparison, a tube of length 16 mm, internal radius $r_\text {in}=4$ mm and external radius $r_\text {out}=6$ mm has a dissection of length 4 mm at a radius of $r=5$ mm. The inner tube surface and tear faces are loaded with a pressure $p=0.1$ kPa, while the outer tube surface is stress-free. The results, given in Fig. 5, show good agreement between those generated by the FEAP program and the method presented here, with a difference of less than 4% in the maximum radial displacement on the tear faces. The greatest relative differences are in the axial displacement of points on the tear faces, due to the tube having finite length in the finite-element computation, and to the method presented here being a linearisation of the full problem.

## Results

**Fig. 6:**
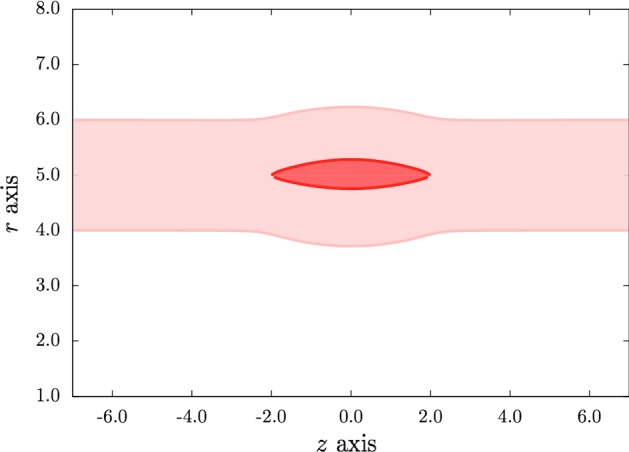
Section through the wall of the cylindrical artery showing the profile of the axisymmetric dissection for $\beta =60^{\circ }$, $\alpha =45^{\circ }$, and $\lambda =1.1$. All lengths are in mm. (Other parameter values are specified in the text)

**Fig. 7:**
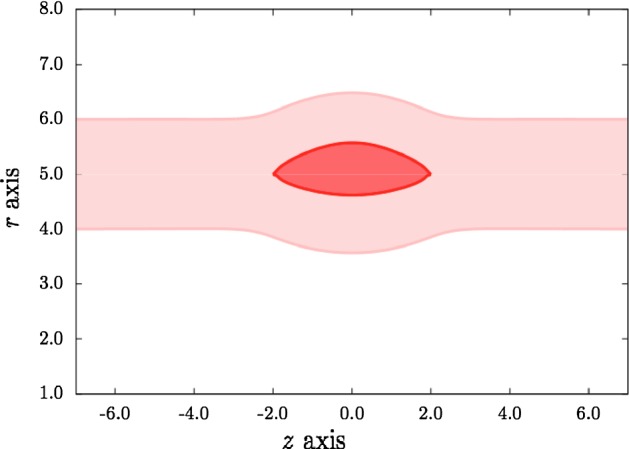
Dissection profile showing that decreasing the collagen fibre angle widens the tear. Here $\beta =30^{\circ }$, $\alpha =45^{\circ }$ and $\lambda =1.1$. All lengths are in mm

**Fig. 8:**
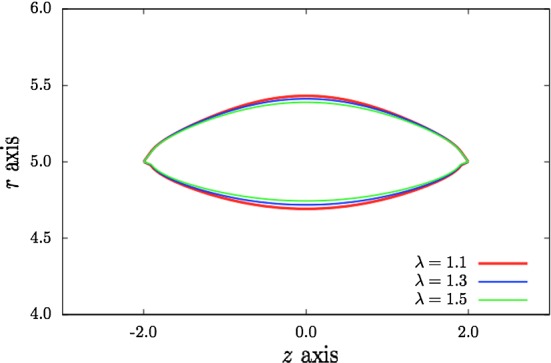
Comparison of dissection profiles for different values of the axial pre-stretch $\lambda$ when $\alpha =30^\circ$ and $\beta =30^\circ$. All lengths are in mm

The following results are for dissection profiles with geometric parameters $r_\text {in}=4\,\mathrm {mm}$, $r_c=5\,\mathrm {mm}$, $r_\text {out}=6\,\mathrm {mm}$ and $R_i=3.9\,\mathrm {mm}$, material parameters $k_1=2.3632\,\mathrm {kPa}$, $k_2=0.8393$, and $c=3\,\mathrm {kPa}$, and outer boundary pressure $P_\text {ext}=0\,\mathrm {kPa}$. This choice of geometric parameter values ensures that there are no wrinkles on the inner boundary when the artery is deformed from the stress-free reference configuration $\Omega _0$ to the current $\Omega$ with residual stress and axial stretch (see Fig. 3), and are typical of a large artery. The material parameters are those used in Figure 14 of Holzapfel et al. (2000) for a rabbit carotid artery. In Figs. 6, 7 and 8, we give examples of the effects of varying the axial pre-stretch $\lambda$, the opening angle $\alpha$ that specifies the residual stress, and the fibre angle $\beta$.

A typical dissection profile is shown in Fig. 6 for an opening angle $\alpha =45^{\circ }$, a collagen fibre angle $\beta = 60^{\circ }$ (see Fig. 2) and an axial pre-stretch $\lambda = 1.1$. The expansion of the dissection creates bulges of approximately the same size and shape on both the inner and outer boundaries of the vessel wall, because the dissection is equidistant from the radial boundaries in the current configuration.

In Fig. 7, the fibre angle has been reduced to 30$^{\circ }$ so that the collagen fibres are aligned more circumferentially than in Fig. 6. The dissection is wider and protrudes further in to the lumen, showing the importance of the axial component of the fibres in reducing a dissection in a pre-stretched artery. A similar result was found by Wang et al. (2015) for a pressurised tear in a 2D strip.

The fibre angle $\beta = 30^{\circ }$ in Fig. 8, as in Fig. 7, and we show dissection profiles for three values of the axial pre-stretch $\lambda$. Increasing $\lambda$ is shown to lead to a narrowing of the tear as the increasing tension in the collagen fibres resists the deformation. Here the opening angle is $\alpha = 30^{\circ }$ so that, by comparing the shape of the dissection for $\lambda = 1.1$ with that in Fig. 7 for which $\alpha = 45^{\circ }$, we see that a smaller opening angle (i.e. a reduced residual stress) leads to a narrower dissection. This result has been confirmed for other parameter values by Li (2013), in which it was also shown that decreasing $r_{\text {in}}$ increases the width of the dissection.

## Incremental blood pressure changes

We treat a small increase in blood pressure in the lumen and tear as an incremental change in the pressure ${\dot{P}}$ on both the inner radius of the tube and the faces of the dissection. The traction $(T_r(z),T_z(z))$ and displacement (*u*, *w*) become

41 $$\begin{aligned} {T}_{r}(z)&=\int {{{\dot{S}}}_{0rr}^u\left( z-s,r\right) }U(s)\,ds + \int {{{\dot{S}}}_{0rr}^w\left( z-s,r\right) }W(s)\,ds +{{\dot{S}}}_{0rr}^{P}{\dot{P}}, \end{aligned}$$

42 $$\begin{aligned} {T}_{z}(z)&=\int {{{\dot{S}}}_{0rz}^u\left( z-s,r\right) }U(s)\,ds + \int {{{\dot{S}}}_{0rz}^w\left( z-s,r\right) }W(s)\,ds, \end{aligned}$$

43 $$\begin{aligned} u&=\int u^u(z-s,r)U(s)\,ds + \int u^w(z-s,r)W(s)\,ds+u^{P}{\dot{P}},\end{aligned}$$

44 $$\begin{aligned} w&=\int w^u(z-s,r)U(s)\,ds + \int w^w(z-s,r)W(s)\,ds. \end{aligned}$$

The quantities (*u*, *w*) and ${{\dot{S}}}_{0rr}^u, {{\dot{S}}}_{0rz}^u, {{\dot{S}}}_{0rr}^w, {{\dot{S}}}_{0rz}^w$, and $u^u$, $w^u, u^w, w^w$ are defined in (23) and (28). We must additionally obtain ${{\dot{S}}}_{0rr}^{P}$ and $u^{P}$, which are the radial components of displacement and stress at the tear face as a result of applying the incremental pressure.

The displacement field due to an incremental change to the inner pressure is radial. The equilibrium equations in (6) and (8), and the incompressibility condition, are

45 $$\begin{aligned} \frac{dq}{dr}=A(r),\quad \frac{d{\dot{q}}}{dr}=\frac{B(r)}{\mu }u, \quad \frac{du}{dr}=-\frac{u}{r}, \end{aligned}$$

where *q* is the hydrostatic pressure, *A*(*r*) and *B*(*r*) are known functions of *r* and *k* (related to the moduli of the material) and of the material and deformation parameters (see Li 2013 and the Supplementary Material). As in Sect. 4, we solve these equations numerically by splitting the domain $[r_\text {in},r_\text {out}]$ into the two regions $[r_\text {in},r_\text {c}]$ and $[r_\text {c},r_\text {out}]$ and transforming both onto the interval $R=[0,1]$ so that $R=0$ corresponds to the inner/outer boundary of the tube and $R=1$ corresponds to the tear face (approached from either the inner or outer section).

We define

$$\begin{aligned} Y_1&=q, ~Y_2=u,&Y_3 ={\dot{q}}&\quad \text {in region 1}, \\ Y_4&=q, ~Y_5=u,&Y_6={\dot{q}}&\quad \text {in region 2}, \\ Y_{a1}&=q, ~Y_{a2}=u,&Y_{a3} = {\dot{q}}&\quad \text {at the inner boundary}, \\ Y_{a4}&=q, ~Y_{a5}=u,&Y_{a6} = {\dot{q}}&\quad \text {at the outer boundary}, \\ Y_{b1}&=q, ~Y_{b2}=u,&Y_{b3} = {\dot{q}}&\quad \text {at the lower dissection face}, \\ Y_{b4}&=q, ~Y_{b5}=u,&Y_{b6} = {\dot{q}}&\quad \text {at the upper dissection face}. \end{aligned}$$

Then from (45), in region 1,

46 $$\begin{aligned} \frac{dY_1}{dr_1}=A(r_1),\quad \frac{dY_2}{dr_1}=-\frac{u}{r_1}, \quad \frac{dY_3}{dr_1}=\frac{B(r_1)}{\mu }u, \end{aligned}$$

where $r_1=r_\text {in}+R(r_c-r_\text {in})$, whilst in region 2,

47 $$\begin{aligned} \frac{dY_4}{dr_2}=A(r_2),\quad \frac{dY_5}{dr_2}=-\frac{u}{r_2}, \quad \frac{dY_6}{dr_2}=\frac{B(r_2)}{\mu }u, \end{aligned}$$

where $$r_2=r_\text {out}+R(r_c-r_\text {out})$$.

Writing the boundary conditions in (6) and (8) in components, yields

$$\begin{aligned}&\sigma _{rr}-P_\text {ext}=0\quad \text {at} \quad r=r_\text {out},\\&{\dot{S}}_{rr}-P_\text {in}\frac{du}{dr}+{\dot{P}}=0\quad \text {at} \quad r=r_\text {in},\\&{\dot{S}}_{rr}-P_\text {ext}\frac{du}{dr}=0\quad \text {at} \quad r=r_\text {out}, \end{aligned}$$

leading to

$$\begin{aligned} \mu \, \left( { a_r} \left( { r_\text {out}} \right) \right) ^{2} -\mu \,Y_{a1} -2/3\,{F_1} \left( { r_\text {out}} \right) {I_4} \left( { r_\text {out}} \right) -2/3\,{F_2} \left( { r_\text {out}} \right) {I_6} \left( { r_\text {out}} \right) -{ P_\text {ext}}&=0,\\ a(r_\text {in})u(r_\text {in})-\mu {\dot{q}}(r_\text {in})+{\dot{P}}=0 \quad \text {and} \quad b(r_\text {out})u(r_\text {out})-\mu {\dot{q}}(r_\text {out})&=0, \end{aligned}$$

where *a*(*r*), *b*(*r*) are known functions of *r*, *k*, material parameters, and deformation parameters (see Li 2013 and the Supplementary Material). The jump conditions are

$$\begin{aligned} Y_{b4}-Y_{b1}=0,\quad Y_{b5}-Y_{b2}=0,\quad Y_{b6}-Y_{b3}=0\quad \text {at} \quad r=r_c. \end{aligned}$$

Given all the ODEs, boundary conditions and jump conditions, the Matlab routine bvp4c is used to calculate $q, u, {\dot{q}}$ in regions 1 and 2, and ${{\dot{S}}}_{0rr}^{P}=S_1(r)u(r)-{\dot{q}}(r)$, in which $S_1(r)$ is a known function of *r* (Li 2013). Hence, once the traction $(T_r,\,T_z)$ is given, we are able to obtain the displacement $(u,\,w)$ for the upper and lower dissection faces, and the inner and outer boundaries. The traction on the tear is

$$\begin{aligned} {T_{r}}=\sigma (r_\text {in})-\sigma (r_{c})-{\dot{P}} \quad \hbox {and}\quad {T_{z}}=0. \end{aligned}$$

**Fig. 9:**
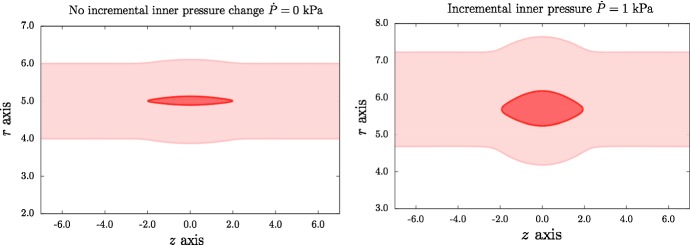
Comparison of dissection profiles when the incremental blood pressure is ${\dot{P}}=0$ and 1 kPa, with $\beta =60^{\circ }$, $\alpha =0^{\circ }$ and $\lambda =1.1$. All lengths are in mm

**Fig. 10:**
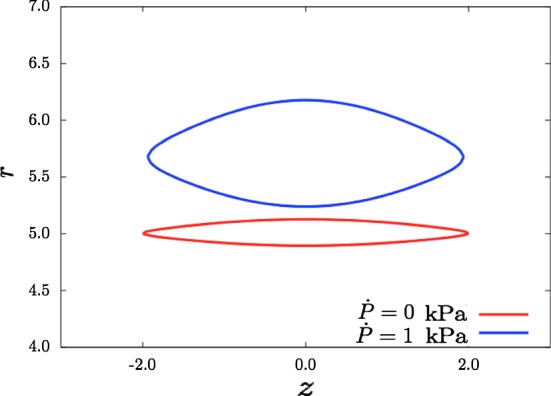
Profiles for the dissection faces when the incremental blood pressure is ${\dot{P}}=0$ and ${\dot{P}}=1$ kPa, with $\beta =60^{\circ }$, $\alpha =0^{\circ }$ and $\lambda =1.1$. All lengths are in mm

Figures 9 and 10 show that the opening of the tear widens as the incremental inner pressure increases. From Fig. 9, we find that tear and the arterial wall are pushed away from original location when the incremental inner pressure is ${\dot{P}}=1$ kPa, and the tear widens and is displaced towards the inner boundary.

## Conclusions

We have derived and solved a mathematical model for the incremental deformation of a prescribed axisymmetric tear in an idealized large artery, which is a single-layer, thick-walled nonlinear incompressible axisymmetric hyperelastic tube with residual stress and two families of collagen fibres, all described by an HGO strain-energy function (Holzapfel et al. 2000).

From the results, we conclude that for the HGO material, subject to residual stress and axial pre-stretch, a dissection is widened by increasing the blood pressure ${\dot{P}}$ within the lumen and the dissection, decreasing the radius of the lumen $r_{\text {in}}$, decreasing the fibre angle $\beta$, by decreasing the axial pre-stretch $\lambda$, and increasing the opening angle $\alpha$. The dependence on $\alpha$ implies that higher values of residual stress promote wider dissections which is unexpected and counters the effect of increasing the axial pre-stretch. We also note that displacements at the inner surface of the wall are greater than at the outer surface.

Immediate extensions of this work are to consider the full two-layer model of (Holzapfel et al. 2000), and to include a multi-cut model in which the residual stress is described by two or more cuts of the unloaded tube (Omens et al. 2003). An important limitation is that the model does not address the propagation of a dissection. To do this, a macroscopic description of the tearing, such as a cohesive zone model, is required, and the direction of tear propagation will be constrained by the layering in the wall of the artery.

We conclude by noting that this work gives insights into the key mechanisms that govern the shape of a tear in the arterial wall and can be used to inform and validate future fully-numerical models in realistic geometries.

## Electronic supplementary material

Below is the link to the electronic supplementary material.

Supplementary material 1 (pdf 177 KB)
