# Supplementary material for: An incremental deformation model of arterial dissection
Source: J Math Biol. 2018 Nov 19;78(5):1277–98. doi: 10.1007/s00285-018-1309-8 (PMC6453878; doi:10.1007/s00285-018-1309-8)
Supplement: Supplementary file 1 — Supplementary material 1 (pdf 177 KB) [file 285_2018_1309_MOESM1_ESM.pdf]

Noname manuscript No.
(will be inserted by the editor)

Supplementary Material for *An Incremental Deformation Model of Arterial Dissection*

Beibei Li, Steven M. Roper, Lei Wang, Xiaoyu Luo and N.A. Hill*

the date of receipt and acceptance should be inserted later

This Supplementary Material contains the full expansions for a number of the parameters used in the paper.

Address(es) of author(s) should be given

1 Coefficients for Jump in 'w'

The coefficients for Jump in 'w' are the parameters used Section 3.2 of the paper.

$$
\begin{aligned}
A_1 = {} & \frac{\mu\,(a_z(r))^2 g}{r} + 2/3\,\frac{F_1(r)\,g\,(\sin(\beta))^2 (a_z(r))^2}{r} \\
& - 4\,\frac{P_1(r)(\cos(\beta))^2 (a_\theta(r))^2 g\,(\sin(\beta))^2 (a_z(r))^2}{r} + 4/3\,\frac{P_1(r)(\cos(\beta))^4 (a_\theta(r))^4 g}{r} \\
& + \frac{g\mu\,(a_\theta(r))^2}{r} - \frac{g\mu\,(a_r(r))^2}{r} - \frac{\mu\,(a_r(r))^2}{g r^3} + 10/3\,\frac{F_1(r)(\cos(\beta))^2 (a_\theta(r))^2 g}{r} \\
& + 2/3\,\frac{F_2(r)\,g\,(\sin(\beta))^2 (a_z(r))^2}{r} + 10/3\,\frac{F_2(r)(\cos(\beta))^2 (a_\theta(r))^2 g}{r} \\
& - 4\,\frac{P_2(r)(\cos(\beta))^2 (a_\theta(r))^2 g\,(\sin(\beta))^2 (a_z(r))^2}{r} + 4/3\,\frac{P_2(r)(\cos(\beta))^4 (a_\theta(r))^4 g}{r} \\
& + 8/3\,\frac{P_1(r)\,g\,(\sin(\beta))^4 (a_z(r))^4}{r} + 8/3\,\frac{P_2(r)\,g\,(\sin(\beta))^4 (a_z(r))^4}{r} - 2\,g\mu\,a_r(r)\frac{d}{dr}a_r(r) \\
& - 2/3\left(\frac{d}{dr}F_1(r)\right) g\,(\cos(\beta))^2 (a_\theta(r))^2 - 2/3\left(\frac{d}{dr}F_1(r)\right) g\,(\sin(\beta))^2 (a_z(r))^2 \\
& - 2/3\left(\frac{d}{dr}F_2(r)\right) g\,(\cos(\beta))^2 (a_\theta(r))^2 - 2/3\left(\frac{d}{dr}F_2(r)\right) g\,(\sin(\beta))^2 (a_z(r))^2 \\
& + 2/3\,gF_1(r)\frac{d}{dr}I_4(r) + 2/3\,g\left(\frac{d}{dr}F_1(r)\right) I_4(r) + 2/3\,gF_2(r)\frac{d}{dr}I_6(r) \\
& + 2/3\,g\left(\frac{d}{dr}F_2(r)\right) I_6(r) + 2\,\frac{\mu\,a_r(r)\frac{d}{dr}a_r(r)}{g r^2} \\
& - 4/3\,F_1(r)\,g\,(\cos(\beta))^2 a_\theta(r)\frac{d}{dr}a_\theta(r) - 4/3\,F_1(r)\,g\,(\sin(\beta))^2 a_z(r)\frac{d}{dr}a_z(r) \\
& - 4/3\,F_2(r)\,g\,(\cos(\beta))^2 a_\theta(r)\frac{d}{dr}a_\theta(r) - 4/3\,F_2(r)\,g\,(\sin(\beta))^2 a_z(r)\frac{d}{dr}a_z(r)
\end{aligned}
$$

$$
\begin{aligned}
B_1 = {} & \mu\,(a_z(r))^2 g + 8/3\,P_1(r)\,g\,(\sin(\beta))^4 (a_z(r))^4 \\
& - 4/3\,P_1(r)\,g\,(\sin(\beta))^2 (a_z(r))^2 (\cos(\beta))^2 (a_\theta(r))^2 \\
& + 8/3\,P_2(r)\,g\,(\sin(\beta))^4 (a_z(r))^4 - 4/3\,P_2(r)(\cos(\beta))^2 (a_\theta(r))^2 g\,(\sin(\beta))^2 (a_z(r))^2 \\
& + \frac{\mu\,(a_r(r))^2}{g r^2} - 2\,\frac{\mu\,a_r(r)\frac{d}{dr}a_r(r)}{g r} + 2/3\,F_1(r)\,g\,(\sin(\beta))^2 (a_z(r))^2 \\
& + 2/3\,F_2(r)\,g\,(\sin(\beta))^2 (a_z(r))^2
\end{aligned}
$$

$$C_1 = -2\,\frac{\mu\,a_r(r)\frac{d}{dr}a_r(r)}{g} - 2\,\frac{\mu\,(a_r(r))^2}{g r}$$

$$D_1 = -\frac{\mu\,(a_r(r))^2}{g}$$

$$E_1 = \mu g$$

$$
\begin{aligned}
A_2 = {} & 8/3\,\frac{F_2(r)(\cos(\beta))^2 a_\theta(r)\frac{d}{dr}a_\theta(r)}{r} + \mu\,(a_z(r))^2 g^2 \\
& + 4/3\,\frac{\left(\frac{d}{dr}F_2(r)\right)(\cos(\beta))^2(a_\theta(r))^2}{r} - 4/3\,\frac{\left(\frac{d}{dr}F_2(r)\right)(\sin(\beta))^2(a_z(r))^2}{r} \\
& + 2/3\,\frac{F_2(r)(\cos(\beta))^2(a_\theta(r))^2}{r^2} + 4/3\,\frac{F_2(r)(\sin(\beta))^2(a_z(r))^2}{r^2} \\
& + 4/3\,\frac{\left(\frac{d}{dr}F_1(r)\right)(\cos(\beta))^2(a_\theta(r))^2}{r} - 4/3\,\frac{\left(\frac{d}{dr}F_1(r)\right)(\sin(\beta))^2(a_z(r))^2}{r} \\
& + 4/3\,\frac{\left(\frac{d}{dr}P_1(r)\right)(\cos(\beta))^4(a_\theta(r))^4}{r} - 4/3\,\frac{\left(\frac{d}{dr}P_1(r)\right)(\sin(\beta))^4(a_z(r))^4}{r} \\
& + 4/3\,\frac{\left(\frac{d}{dr}P_2(r)\right)(\cos(\beta))^4(a_\theta(r))^4}{r} - 4/3\,\frac{\left(\frac{d}{dr}P_2(r)\right)(\sin(\beta))^4(a_z(r))^4}{r} \\
& + 4/3\,\frac{P_2(r)(\sin(\beta))^4(a_z(r))^4}{r^2} + 8/3\,\frac{P_2(r)(\cos(\beta))^4(a_\theta(r))^4}{r^2} \\
& + 2/3\,\frac{F_1(r)(\cos(\beta))^2(a_\theta(r))^2}{r^2} + 4/3\,\frac{F_1(r)(\sin(\beta))^2(a_z(r))^2}{r^2} \\
& + 4/3\,\frac{P_1(r)(\sin(\beta))^4(a_z(r))^4}{r^2} + 2\,F_1(r)(\sin(\beta))^2(a_z(r))^2 g^2 \\
& + 2\,F_2(r)(\sin(\beta))^2(a_z(r))^2 g^2 + 8/3\,\frac{F_1(r)(\cos(\beta))^2 a_\theta(r)\frac{d}{dr}a_\theta(r)}{r} \\
& - 8/3\,\frac{F_1(r)(\sin(\beta))^2 a_z(r)\frac{d}{dr}a_z(r)}{r} + 16/3\,\frac{P1(r)(\cos(\beta))^4(a_\theta(r))^3\frac{d}{dr}a_\theta(r)}{r} \\
& - 16/3\,\frac{P_1(r)(\sin(\beta))^4(a_z(r))^3\frac{d}{dr}a_z(r)}{r} - 8/3\,\frac{F_2(r)(\sin(\beta))^2 a_z(r)\frac{d}{dr}a_z(r)}{r} \\
& - 4\,\frac{P_2(r)(\sin(\beta))^2(a_z(r))^2(\cos(\beta))^2(a_\theta(r))^2}{r^2} \\
& - 4\,\frac{P_1(r)(\sin(\beta))^2(a_z(r))^2(\cos(\beta))^2(a_\theta(r))^2}{r^2} \\
& - 16/3\,\frac{P_2(r)(\sin(\beta))^4(a_z(r))^3\frac{d}{dr}a_z(r)}{r} \\
& + 16/3\,\frac{P_2(r)(\cos(\beta))^4(a_\theta(r))^3\frac{d}{dr}a_\theta(r)}{r} + \frac{\mu\,(a_\theta(r))^2}{r^2} \\
& + 8/3\,\frac{P_1(r)(\cos(\beta))^4(a_\theta(r))^4}{r^2}
\end{aligned}
$$

$$
\begin{aligned}
B_2 = & -4/3\left(\frac{d}{dr}P_2(r)\right)(\sin(\beta))^4(a_z(r))^4-2\left(\frac{d}{dr}F_2(r)\right)(\sin(\beta))^2(a_z(r))^2\\
& -16/3\,P_1(r)(\sin(\beta))^4(a_z(r))^3\frac{d}{dr}a_z(r)-4\,F_2(r)(\sin(\beta))^2a_z(r)\frac{d}{dr}a_z(r)\\
& -2\left(\frac{d}{dr}F_1(r)\right)(\sin(\beta))^2(a_z(r))^2-16/3\,P_2(r)(\sin(\beta))^4(a_z(r))^3\frac{d}{dr}a_z(r)\\
& -4\,F_1(r)(\sin(\beta))^2a_z(r)\frac{d}{dr}a_z(r)-4/3\left(\frac{d}{dr}P_1(r)\right)(\sin(\beta))^4(a_z(r))^4\\
& -2/3\left(\frac{d}{dr}F_1(r)\right)(\cos(\beta))^2(a_\theta(r))^2+2/3\left(\frac{d}{dr}F_1(r)\right)I_4(r)+2/3\,F_2(r)\frac{d}{dr}I_6(r)\\
& +2/3\,F_1(r)\frac{d}{dr}I_4(r)+2/3\left(\frac{d}{dr}F_2(r)\right)I_6(r)-2\,\frac{\mu\,(a_r(r))^2}{r}\\
& -4/3\,F_1(r)(\cos(\beta))^2a_\theta(r)\frac{d}{dr}a_\theta(r)-4/3\,F_2(r)(\cos(\beta))^2a_\theta(r)\frac{d}{dr}a_\theta(r)\\
& +10/3\,\frac{F_1(r)(\cos(\beta))^2(a_\theta(r))^2}{r}+10/3\,\frac{F_2(r)(\cos(\beta))^2(a_\theta(r))^2}{r}\\
& -4/3\,\frac{F_1(r)(\sin(\beta))^2(a_z(r))^2}{r}-2/3\left(\frac{d}{dr}F_2(r)\right)(\cos(\beta))^2(a_\theta(r))^2\\
& +\frac{\mu\,(a_\theta(r))^2}{r}-4\,\mu\,a_r(r)\frac{d}{dr}a_r(r)-4\,\frac{P_1(r)(\sin(\beta))^2(a_z(r))^2(\cos(\beta))^2(a_\theta(r))^2}{r}\\
& -8/3\,P_2(r)(\sin(\beta))^2(a_z(r))^2(\cos(\beta))^2a_\theta(r)\frac{d}{dr}a_\theta(r)\\
& -8/3\,P_1(r)(\sin(\beta))^2a_z(r)\left(\frac{d}{dr}a_z(r)\right)(\cos(\beta))^2(a_\theta(r))^2\\
& -4/3\left(\frac{d}{dr}P_1(r)\right)(\sin(\beta))^2(a_z(r))^2(\cos(\beta))^2(a_\theta(r))^2\\
& -8/3\,P_1(r)(\sin(\beta))^2(a_z(r))^2(\cos(\beta))^2a_\theta(r)\frac{d}{dr}a_\theta(r)\\
& -4/3\left(\frac{d}{dr}P_2(r)\right)(\sin(\beta))^2(a_z(r))^2(\cos(\beta))^2(a_\theta(r))^2\\
& -8/3\,P_2(r)(\sin(\beta))^2a_z(r)\left(\frac{d}{dr}a_z(r)\right)(\cos(\beta))^2(a_\theta(r))^2\\
& -4\,\frac{P_2(r)(\sin(\beta))^2(a_z(r))^2(\cos(\beta))^2(a_\theta(r))^2}{r}\\
& -4/3\,\frac{F_2(r)(\sin(\beta))^2(a_z(r))^2}{r}-4/3\,\frac{P_2(r)(\sin(\beta))^4(a_z(r))^4}{r}\\
& +4/3\,\frac{P_2(r)(\cos(\beta))^4(a_\theta(r))^4}{r}-4/3\,\frac{P_1(r)(\sin(\beta))^4(a_z(r))^4}{r}\\
& +4/3\,\frac{P_1(r)(\cos(\beta))^4(a_\theta(r))^4}{r}
\end{aligned}
$$

$$
\begin{aligned}
C_2 = & -4/3\,P_2(r)(\sin(\beta))^2(a_z(r))^2(\cos(\beta))^2(a_\theta(r))^2-4/3\,F_1(r)(\sin(\beta))^2(a_z(r))^2\\
& -4/3\,F_2(r)(\sin(\beta))^2(a_z(r))^2-4/3\,P_1(r)(\sin(\beta))^2(a_z(r))^2(\cos(\beta))^2(a_\theta(r))^2\\
& -\mu\,(a_r(r))^2-4/3\,P_2(r)(\sin(\beta))^4(a_z(r))^4-4/3\,P_1(r)(\sin(\beta))^4(a_z(r))^4
\end{aligned}
$$

$$D_2 = \mu$$

$$\begin{aligned} a_1 = {} & 4/3\,\frac{P_1(r_{in})(\cos(\beta))^4(a_\theta(r_{in}))^4}{r_{in}} - 4/3\,\frac{F_1(r_{in})(\sin(\beta))^2(a_z(r_{in}))^2}{r_{in}} \\ & - 4/3\,\frac{P_1(r_{in})(\sin(\beta))^4(a_z(r_{in}))^4}{r_{in}} - 4/3\,\frac{F_2(r_{in})(\sin(\beta))^2(a_z(r_{in}))^2}{r_{in}} \\ & + 4/3\,\frac{F_2(r_{in})(\cos(\beta))^2(a_\theta(r_{in}))^2}{r_{in}} + 4/3\,\frac{F_1(r_{in})(\cos(\beta))^2(a_\theta(r_{in}))^2}{r_{in}} \\ & - 4/3\,\frac{P_2(r_{in})(\sin(\beta))^4(a_z(r_{in}))^4}{r_{in}} + 4/3\,\frac{P_2(r_{in})(\cos(\beta))^4(a_\theta(r_{in}))^4}{r_{in}} \end{aligned}$$

$$\begin{aligned} a_2 = {} & -4/3\,P_2(r_{in})(\sin(\beta))^4(a_z(r_{in}))^4 - 2/3\,F_1(r_{in})\,I_4(r_{in}) - 2/3\,F_2(r_{in})\,I_6(r_{in}) \\ & - 2/3\,F_2(r_{in})(\cos(\beta))^2(a_\theta(r_{in}))^2 - 2\,F_1(r_{in})(\sin(\beta))^2(a_z(r_{in}))^2 - 2\,\mu\,q(r_{in}) \\ & - 4/3\,P_1(r_{in})(\sin(\beta))^2(a_z(r_{in}))^2(\cos(\beta))^2(a_\theta(r_{in}))^2 - 2\,F_2(r_{in})(\sin(\beta))^2(a_z(r_{in}))^2 \\ & - 2/3\,F_1(r_{in})(\cos(\beta))^2(a_\theta(r_{in}))^2 - 4/3\,P_2(r_{in})(\sin(\beta))^2(a_z(r_{in}))^2(\cos(\beta))^2(a_\theta(r_{in}))^2 \\ & - 4/3\,P_1(r_{in})(\sin(\beta))^4(a_z(r_{in}))^4 \end{aligned}$$

$$a_4 = \mu$$

$$\begin{aligned} b_1 = {} & -2\,g\mu\,q(r_{in}) + \frac{\mu\,(a_r(r_{in}))^2}{g{r_{in}}^2} - 2/3\,F_1(r_{in})\,g\,(\cos(\beta))^2(a_\theta(r_{in}))^2 \\ & - 2/3\,F_1(r_{in})\,g\,(\sin(\beta))^2(a_z(r_{in}))^2 - 2/3\,F_2(r_{in})\,g\,(\cos(\beta))^2(a_\theta(r_{in}))^2 \\ & - 2/3\,F_2(r_{in})\,g\,(\sin(\beta))^2(a_z(r_{in}))^2 + g\mu\,(a_r(r_{in}))^2 \\ & - 2/3\,gF_1(r_{in})\,I_4(r_{in}) - 2/3\,gF_2(r_{in})\,I_6(r_{in}) \end{aligned}$$

$$b_2 = -\frac{\mu\,(a_r(r_{in}))^2}{gr_{in}}$$

$$b_3 = -\frac{\mu\,(a_r(r_{in}))^2}{g}$$

$$\begin{aligned} c_5 = {} & 4/3\,\frac{F_2(r_{out})(\cos(\beta))^2(a_\theta(r_{out}))^2}{r_{out}} - 4/3\,\frac{F_2(r_{out})(\sin(\beta))^2(a_z(r_{out}))^2}{r_{out}} \\ & - 4/3\,\frac{P_1(r_{out})(\sin(\beta))^4(a_z(r_{out}))^4}{r_{out}} - 4/3\,\frac{P_2(r_{out})(\sin(\beta))^4(a_z(r_{out}))^4}{r_{out}} \\ & + 4/3\,\frac{F_1(r_{out})(\cos(\beta))^2(a_\theta(r_{out}))^2}{r_{out}} + 4/3\,\frac{P_1(r_{out})(\cos(\beta))^4(a_\theta(r_{out}))^4}{r_{out}} \\ & + 4/3\,\frac{P_2(r_{out})(\cos(\beta))^4(a_\theta(r_{out}))^4}{r_{out}} - 4/3\,\frac{F_1(r_{out})(\sin(\beta))^2(a_z(r_{out}))^2}{r_{out}} \end{aligned}$$

$$\begin{aligned}c_6 = &-\mu\,(a_r(r_{out}))^2 - 4/3\,P_2(r_{out})(\sin(\beta))^2(a_z(r_{out}))^2(\cos(\beta))^2(a_\theta(r_{out}))^2\\ &-\mu\,q(r_{out}) - 2/3\,F_1(r_{out})(\cos(\beta))^2(a_\theta(r_{out}))^2 + P_{ext} - 4/3\,P_1(r_{out})(\sin(\beta))^4(a_z(r_{out}))^4\\ &-2/3\,F_2(r_{out})(\cos(\beta))^2(a_\theta(r_{out}))^2 - 4/3\,P_1(r_{out})(\sin(\beta))^2(a_z(r_{out}))^2(\cos(\beta))^2(a_\theta(r_{out}))^2\\ &-4/3\,P_2(r_{out})(\sin(\beta))^4(a_z(r_{out}))^4 - 2\,F_1(r_{out})(\sin(\beta))^2(a_z(r_{out}))^2\\ &-2\,F_2(r_{out})(\sin(\beta))^2(a_z(r_{out}))^2\end{aligned}$$

$$c_8 = \mu$$

$$\begin{aligned}d_5 = &-\mu\,q(r_{out})\,g + \frac{\mu\,(a_r(r_{out}))^2}{g{r_{out}}^2} - 2/3\,F_1(r_{out})\,g(\cos(\beta))^2(a_\theta(r_{out}))^2\\ &-2/3\,F_1(r_{out})\,g(\sin(\beta))^2(a_z(r_{out}))^2 - 2/3\,F_2(r_{out})\,g(\cos(\beta))^2(a_\theta(r_{out}))^2\\ &-2/3\,F_2(r_{out})\,g(\sin(\beta))^2(a_z(r_{out}))^2 + P_{ext}\,g\end{aligned}$$

$$d_6 = -\frac{\mu\,(a_r(r_{out}))^2}{g r_{out}}$$

$$d_7 = -\frac{\mu\,(a_r(r_{out}))^2}{g}$$

$$\begin{aligned}s_{wr1}(r) = &\,4/3\,\frac{P_2(r)(\cos(\beta))^4(a_\theta(r))^4}{r} + 4/3\,\frac{P_1(r)(\cos(\beta))^4(a_\theta(r))^4}{r}\\ &-4/3\,\frac{P_2(r)(\sin(\beta))^4(a_z(r))^4}{r} - 4/3\,\frac{P_1(r)(\sin(\beta))^4(a_z(r))^4}{r}\\ &-4/3\,\frac{F_1(r)(\sin(\beta))^2(a_z(r))^2}{r} + 4/3\,\frac{F_2(r)(\cos(\beta))^2(a_\theta(r))^2}{r}\\ &+4/3\,\frac{F_1(r)(\cos(\beta))^2(a_\theta(r))^2}{r} - 4/3\,\frac{F_2(r)(\sin(\beta))^2(a_z(r))^2}{r}\end{aligned}$$

$$\begin{aligned}s_{wr2}(r) = &-4/3\,P_1(r)(\sin(\beta))^4(a_z(r))^4\\ &-4/3\,P_2(r)(\sin(\beta))^2(a_z(r))^2(\cos(\beta))^2(a_\theta(r))^2\\ &-4/3\,P_2(r)(\sin(\beta))^4(a_z(r))^4 - 2\,F_1(r)(\sin(\beta))^2(a_z(r))^2\\ &-\mu\,(a_r(r))^2 - 4/3\,P_1(r)(\sin(\beta))^2(a_z(r))^2(\cos(\beta))^2(a_\theta(r))^2\\ &-\mu\,q(r) - 2\,F_2(r)(\sin(\beta))^2(a_z(r))^2 - 2/3\,F_2(r)(\cos(\beta))^2(a_\theta(r))^2\\ &-2/3\,F_1(r)(\cos(\beta))^2(a_\theta(r))^2\end{aligned}$$

$$\begin{aligned}s_{uz1}(r) = &-\mu\,q(r)g + \frac{\mu\,(a_r(r))^2}{gr^2} - 2/3\,F_1(r)\,g(\cos(\beta))^2(a_\theta(r))^2\\ &-2/3\,F_1(r)\,g(\sin(\beta))^2(a_z(r))^2 - 2/3\,F_2(r)\,g(\cos(\beta))^2(a_\theta(r))^2\\ &-2/3\,F_2(r)\,g(\sin(\beta))^2(a_z(r))^2\end{aligned}$$

$$s_{uz2}(r) = -\frac{\mu\,(a_r(r))^2}{gr}$$

$$s_{uz3}(r) = -\frac{\mu\,(a_r(r))^2}{g}$$

$$\begin{aligned} e_1 = {} & 4/3\,\frac{P_2(r_c)(\cos(\beta))^4(a_\theta(r_c))^4}{r_c} + 4/3\,\frac{F_1(r_c)(\cos(\beta))^2(at(r_c))^2}{r_c} \\ & + 4/3\,\frac{P_1(r_c)(\cos(\beta))^4(a_\theta(r_c))^4}{r_c} - 4/3\,\frac{F_1(r_c)(\sin(\beta))^2(a_z(r_c))^2}{r_c} \\ & + 4/3\,\frac{F_2(r_c)(\cos(\beta))^2(a_\theta(r_c))^2}{r_c} - 4/3\,\frac{P_2(r_c)(\sin(\beta))^4(a_z(r_c))^4}{r_c} \\ & - 4/3\,\frac{F_2(r_c)(\sin(\beta))^2(a_z(r_c))^2}{r_c} - 4/3\,\frac{P_1(r_c)(\sin(\beta))^4(a_z(r_c))^4}{r_c} \end{aligned}$$

$$\begin{aligned} e_2 = {} & -\mu\,Y_{b9} - 2/3\,F_2(r_c)(\cos(\beta))^2(a_\theta(r_c))^2 - 2/3\,F_1(r_c)(\cos(\beta))^2(a_\theta(r_c))^2 \\ & - 2\,F_2(r_c)(\sin(\beta))^2(a_z(r_c))^2 - 4/3\,P_2(r_c)(\sin(\beta))^4(a_z(r_c))^4 - 2\,F_1(r_c)(\sin(\beta))^2(a_z(r_c))^2 \\ & - \mu\,(a_r(r_c))^2 - 4/3\,P_1(r_c)(\sin(\beta))^4(a_z(r_c))^4 \\ & - 4/3\,P_2(r_c)(\sin(\beta))^2(a_z(r_c))^2(\cos(\beta))^2(a_\theta(r_c))^2 \\ & - 4/3\,P_1(r_c)(\sin(\beta))^2(a_z(r_c))^2(\cos(\beta))^2(a_\theta(r_c))^2 \end{aligned}$$

$$e_4 = \mu$$

$$\begin{aligned} e_5 = {} & 4/3\,\frac{P_2(r_c)(\cos(\beta))^4(a_\theta(r_c))^4}{r_c} + 4/3\,\frac{F_1(r_c)(\cos(\beta))^2(at(r_c))^2}{r_c} \\ & + 4/3\,\frac{P_1(r_c)(\cos(\beta))^4(a_\theta(r_c))^4}{r_c} - 4/3\,\frac{F_1(r_c)(\sin(\beta))^2(a_z(r_c))^2}{r_c} \\ & + 4/3\,\frac{F_2(r_c)(\cos(\beta))^2(a_\theta(r_c))^2}{r_c} - 4/3\,\frac{P_2(r_c)(\sin(\beta))^4(a_z(r_c))^4}{r_c} \\ & - 4/3\,\frac{F_2(r_c)(\sin(\beta))^2(a_z(r_c))^2}{r_c} - 4/3\,\frac{P_1(r_c)(\sin(\beta))^4(a_z(r_c))^4}{r_c} \end{aligned}$$

$$\begin{aligned} e_6 = {} & -\mu\,Y_{b10} - 2/3\,F_2(r_c)(\cos(\beta))^2(a_\theta(r_c))^2 - 2/3\,F_1(r_c)(\cos(\beta))^2(a_\theta(r_c))^2 \\ & - 2\,F_2(r_c)(\sin(\beta))^2(a_z(r_c))^2 - 4/3\,P_2(r_c)(\sin(\beta))^4(a_z(r_c))^4 - 2\,F_1(r_c)(\sin(\beta))^2(a_z(r_c))^2 \\ & - \mu\,(a_r(r_c))^2 - 4/3\,P_1(r_c)(\sin(\beta))^4(a_z(r_c))^4 \\ & - 4/3\,P_2(r_c)(\sin(\beta))^2(a_z(r_c))^2(\cos(\beta))^2(a_\theta(r_c))^2 \\ & - 4/3\,P_1(r_c)(\sin(\beta))^2(a_z(r_c))^2(\cos(\beta))^2(a_\theta(r_c))^2 \end{aligned}$$

$$e_8 = \mu$$

$$\begin{aligned} f_1 = {} & -\mu\,Y_{b9}\,g + \frac{\mu\,(a_r(r_c))^2}{g{r_c}^2} - 2/3\,F_1(r_c)\,g\,(\cos(\beta))^2(a_\theta(r_c))^2 - 2/3\,F_1(r_c)\,g\,(\sin(\beta))^2(a_z(r_c))^2 \\ & - 2/3\,F_2(r_c)\,g\,(\cos(\beta))^2(a_\theta(r_c))^2 - 2/3\,F_2(r_c)\,g\,(\sin(\beta))^2(a_z(r_c))^2 \end{aligned}$$

$$f_2 = -\frac{\mu \left(a_r\left(r_c\right)\right)^2}{g r_c}$$

$$f_3 = -\frac{\mu \left(a_r\left(r_c\right)\right)^2}{g}$$

$$f_5 = -\mu Y_{b10} g + \frac{\mu \left(a_r\left(r_c\right)\right)^2}{g {r_c}^2} - 2/3\, F_1\left(r_c\right) g \left(\cos\left(\beta\right)\right)^2 \left(a_\theta\left(r_c\right)\right)^2 - 2/3\, F_1\left(r_c\right) g \left(\sin\left(\beta\right)\right)^2 \left(a_z\left(r_c\right)\right)^2 - 2/3\, F_2\left(r_c\right) g \left(\cos\left(\beta\right)\right)^2 \left(a_\theta\left(r_c\right)\right)^2 - 2/3\, F_2\left(r_c\right) g \left(\sin\left(\beta\right)\right)^2 \left(a_z\left(r_c\right)\right)^2$$

$$f_6 = -\frac{\mu \left(a_r\left(r_c\right)\right)^2}{g r_c}$$

$$f_7 = -\frac{\mu \left(a_r\left(r_c\right)\right)^2}{g}$$

2 Coefficients for Jump in 'u'

The coefficients for Jump in 'u' are the parameters in Section 3.3 of the paper.

$$\begin{aligned}
A_1 = {} & \frac{\mu \left(a_z(r)\right)^2 g}{r} + 2/3\,\frac{F_1(r)\, g \left(\sin(\beta)\right)^2 \left(a_z(r)\right)^2}{r} \\
& - 4\,\frac{P_1(r) \left(\cos(\beta)\right)^2 \left(a_\theta(r)\right)^2 g \left(\sin(\beta)\right)^2 \left(a_z(r)\right)^2}{r} + 4/3\,\frac{P_1(r) \left(\cos(\beta)\right)^4 \left(a_\theta(r)\right)^4 g}{r} \\
& + \frac{g\mu \left(a_\theta(r)\right)^2}{r} - \frac{g\mu \left(a_r(r)\right)^2}{r} - \frac{\mu \left(a_r(r)\right)^2}{g r^3} + 10/3\,\frac{F_1(r) \left(\cos(\beta)\right)^2 \left(a_\theta(r)\right)^2 g}{r} \\
& + 2/3\,\frac{F_2(r)\, g \left(\sin(\beta)\right)^2 \left(a_z(r)\right)^2}{r} + 10/3\,\frac{F_2(r) \left(\cos(\beta)\right)^2 \left(a_\theta(r)\right)^2 g}{r} \\
& - 4\,\frac{P_2(r) \left(\cos(\beta)\right)^2 \left(a_\theta(r)\right)^2 g \left(\sin(\beta)\right)^2 \left(a_z(r)\right)^2}{r} + 4/3\,\frac{P_2(r) \left(\cos(\beta)\right)^4 \left(a_\theta(r)\right)^4 g}{r} \\
& + 8/3\,\frac{P_1(r)\, g \left(\sin(\beta)\right)^4 \left(a_z(r)\right)^4}{r} + 8/3\,\frac{P_2(r)\, g \left(\sin(\beta)\right)^4 \left(a_z(r)\right)^4}{r} - 2\, g\mu\, a_r(r) \frac{d}{dr} a_r(r) \\
& - 2/3 \left(\frac{d}{dr} F_1(r)\right) g \left(\cos(\beta)\right)^2 \left(a_\theta(r)\right)^2 - 2/3 \left(\frac{d}{dr} F_1(r)\right) g \left(\sin(\beta)\right)^2 \left(a_z(r)\right)^2 \\
& - 2/3 \left(\frac{d}{dr} F_2(r)\right) g \left(\cos(\beta)\right)^2 \left(a_\theta(r)\right)^2 - 2/3 \left(\frac{d}{dr} F_2(r)\right) g \left(\sin(\beta)\right)^2 \left(a_z(r)\right)^2 \\
& + 2/3\, g F_1(r) \frac{d}{dr} I_4(r) + 2/3\, g \left(\frac{d}{dr} F_1(r)\right) I_4(r) + 2/3\, g F_2(r) \frac{d}{dr} I_6(r) \\
& + 2/3\, g \left(\frac{d}{dr} F_2(r)\right) I_6(r) + 2\,\frac{\mu\, a_r(r) \frac{d}{dr} a_r(r)}{g r^2} \\
& - 4/3\, F_1(r)\, g \left(\cos(\beta)\right)^2 a_\theta(r) \frac{d}{dr} a_\theta(r) - 4/3\, F_1(r)\, g \left(\sin(\beta)\right)^2 a_z(r) \frac{d}{dr} a_z(r) \\
& - 4/3\, F_2(r)\, g \left(\cos(\beta)\right)^2 a_\theta(r) \frac{d}{dr} a_\theta(r) - 4/3\, F_2(r)\, g \left(\sin(\beta)\right)^2 a_z(r) \frac{d}{dr} a_z(r)
\end{aligned}$$

$$
\begin{aligned}
B_1 = {} & \mu\, \left(a_z\left(r\right)\right)^2 g + 8/3\, P_1\left(r\right) g \left(\sin\left(\beta\right)\right)^4 \left(a_z\left(r\right)\right)^4 \\
& - 4/3\, P_1\left(r\right) g \left(\sin\left(\beta\right)\right)^2 \left(a_z\left(r\right)\right)^2 \left(\cos\left(\beta\right)\right)^2 \left(a_\theta\left(r\right)\right)^2 \\
& + 8/3\, P_2\left(r\right) g \left(\sin\left(\beta\right)\right)^4 \left(a_z\left(r\right)\right)^4 - 4/3\, P_2\left(r\right) \left(\cos\left(\beta\right)\right)^2 \left(a_\theta\left(r\right)\right)^2 g \left(\sin\left(\beta\right)\right)^2 \left(a_z\left(r\right)\right)^2 \\
& + \frac{\mu\, \left(a_r\left(r\right)\right)^2}{g r^2} - 2\, \frac{\mu\, a_r\left(r\right) \frac{d}{dr} a_r\left(r\right)}{g r} + 2/3\, F_1\left(r\right) g \left(\sin\left(\beta\right)\right)^2 \left(a_z\left(r\right)\right)^2 \\
& + 2/3\, F_2\left(r\right) g \left(\sin\left(\beta\right)\right)^2 \left(a_z\left(r\right)\right)^2
\end{aligned}
$$

$$
C_1 = -2\, \frac{\mu\, a_r\left(r\right) \frac{d}{dr} a_r\left(r\right)}{g} - 2\, \frac{\mu\, \left(a_r\left(r\right)\right)^2}{g r}
$$

$$
D_1 = -\frac{\mu\, \left(a_r\left(r\right)\right)^2}{g}
$$

$$
E_1 = \mu g
$$

$$
\begin{aligned}
A_2 = & -4/3\,\frac{P_1(r)(\sin(\beta))^4(a_z(r))^4}{r^2} - 4/3\,\frac{\left(\frac{d}{dr1}P_2(r)\right)(\cos(\beta))^4(a_\theta(r))^4}{r} \\
& + 4/3\,\frac{\left(\frac{d}{dr}P_2(r)\right)(\sin(\beta))^4(a_z(r))^4}{r} - 8/3\,\frac{P_2(r)(\cos(\beta))^4(a_\theta(r))^4}{r^2} \\
& - 4/3\,\frac{P_2(r)(\sin(\beta))^4(a_z(r))^4}{r^2} - 2\,F_1(r)(\sin(\beta))^2(a_z(r))^2 g^2 \\
& - 2\,F_2(r)(\sin(\beta))^2(a_z(r))^2 g^2 - 4/3\,\frac{\left(\frac{d}{dr}F_1(r)\right)(\cos(\beta))^2(a_\theta(r))^2}{r} \\
& + 4/3\,\frac{\left(\frac{d}{dr}F_1(r)\right)(\sin(\beta))^2(a_z(r))^2}{r} - 2/3\,\frac{F_1(r)(\cos(\beta))^2(a_\theta(r))^2}{r^2} \\
& - 4/3\,\frac{F_1(r)(\sin(\beta))^2(a_z(r))^2}{r^2} - 4/3\,\frac{\left(\frac{d}{dr}F_2(r)\right)(\cos(\beta))^2(a_\theta(r))^2}{r} \\
& + 4/3\,\frac{\left(\frac{d}{dr}F_2(r)\right)(\sin(\beta))^2(a_z(r))^2}{r} - 2/3\,\frac{F_2(r)(\cos(\beta))^2(a_\theta(r))^2}{r^2} \\
& - 4/3\,\frac{F_2(r)(\sin(\beta))^2(a_z(r))^2}{r^2} - 16/3\,\frac{P_2(r)(\cos(\beta))^4(a_\theta(r))^3\,\frac{d}{dr}a_\theta(r)}{r} \\
& - \mu\,(a_z(r))^2 g^2 - \frac{\mu\,(a_\theta(r))^2}{r^2} - 4/3\,\frac{\left(\frac{d}{dr}P_1(r)\right)(\cos(\beta))^4(a_\theta(r))^4}{r} \\
& + 4/3\,\frac{\left(\frac{d}{dr}P_1(r)\right)(\sin(\beta))^4(a_z(r))^4}{r} - 8/3\,\frac{P_1(r)(\cos(\beta))^4(a_\theta(r))^4}{r^2} \\
& + 8/3\,\frac{F_1(r)(\sin(\beta))^2 a_z(r)\,\frac{d}{dr}a_z(r)}{r} + 16/3\,\frac{P_2(r)(\sin(\beta))^4(a_z(r))^3\,\frac{d}{dr}a_z(r)}{r} \\
& - 8/3\,\frac{F_2(r)(\cos(\beta))^2 a_\theta(r)\,\frac{d}{dr}a_\theta(r)}{r} + 8/3\,\frac{F_2(r)(\sin(\beta))^2 a_z(r)\,\frac{d}{dr}a_z(r)}{r} \\
& + 4\,\frac{P_1(r)(\cos(\beta))^2(a_\theta(r))^2(\sin(\beta))^2(a_z(r))^2}{r^2} \\
& + 4\,\frac{P_2(r)(\cos(\beta))^2(a_\theta(r))^2(\sin(\beta))^2(a_z(r))^2}{r^2} \\
& - 16/3\,\frac{P_1(r)(\cos(\beta))^4(a_\theta(r))^3\,\frac{d}{dr}a_\theta(r)}{r} \\
& + 16/3\,\frac{P_1(r)(\sin(\beta))^4(a_z(r))^3\,\frac{d}{dr}a_z(r)}{r} \\
& - 8/3\,\frac{F_1(r)(\cos(\beta))^2 a_\theta(r)\,\frac{d}{dr}a_\theta(r)}{r}
\end{aligned}
$$

$$
\begin{aligned}
B_2 =\ & 16/3\, P_2(r) (\sin(\beta))^4 (a_z(r))^3 \frac{d}{dr} a_z(r) + 4/3\, F_2(r) (\cos(\beta))^2 a_\theta(r) \frac{d}{dr} a_\theta(r) \\
& + 4\, F_2(r) (\sin(\beta))^2 a_z(r) \frac{d}{dr} a_z(r) + 2 \frac{\mu\, (a_r(r))^2}{r} + 4/3 \frac{F_1(r) (\sin(\beta))^2 (a_z(r))^2}{r} \\
& + 4/3\, F_1(r) (\cos(\beta))^2 a_\theta(r) \frac{d}{dr} a_\theta(r) \\
& + 8/3\, P_2(r) (\sin(\beta))^2 (a_z(r))^2 (\cos(\beta))^2 a_\theta(r) \frac{d}{dr} a_\theta(r) \\
& + 4/3 \left(\frac{d}{dr} P_1(r)\right) (\sin(\beta))^2 (a_z(r))^2 (\cos(\beta))^2 (a_\theta(r))^2 \\
& + 4 \frac{P_1(r) (\sin(\beta))^2 (a_z(r))^2 (\cos(\beta))^2 (a_\theta(r))^2}{r} \\
& + 4 \frac{P_2(r) (\sin(\beta))^2 (a_z(r))^2 (\cos(\beta))^2 (a_\theta(r))^2}{r} \\
& + 8/3\, P_1(r) (\sin(\beta))^2 a_z(r) \left(\frac{d}{dr} a_z(r)\right) (\cos(\beta))^2 (a_\theta(r))^2 \\
& + 8/3\, P_1(r) (\sin(\beta))^2 (a_z(r))^2 (\cos(\beta))^2 a_\theta(r) \frac{d}{dr} a_\theta(r) \\
& + 4/3 \left(\frac{d}{dr} P_2(r)\right) (\sin(\beta))^2 (a_z(r))^2 (\cos(\beta))^2 (a_\theta(r))^2 \\
& + 8/3\, P_2(r) (\sin(\beta))^2 a_z(r) \left(\frac{d}{dr} a_z(r)\right) (\cos(\beta))^2 (a_\theta(r))^2 \\
& + 2/3 \left(\frac{d}{dr} F_1(r)\right) (\cos(\beta))^2 (a_\theta(r))^2 + 2 \left(\frac{d}{dr} F_2(r)\right) (\sin(\beta))^2 (a_z(r))^2 \\
& + 2/3 \left(\frac{d}{dr} F_2(r)\right) (\cos(\beta))^2 (a_\theta(r))^2 + 4/3 \left(\frac{d}{dr} P_2(r)\right) (\sin(\beta))^4 (a_z(r))^4 \\
& + 4/3 \frac{F_2(r) (\sin(\beta))^2 (a_z(r))^2}{r} - 4/3 \frac{P_2(r) (\cos(\beta))^4 (a_\theta(r))^4}{r} \\
& + 4/3 \frac{P_2(r) (\sin(\beta))^4 (a_z(r))^4}{r} - 10/3 \frac{F_1(r) (\cos(\beta))^2 (a_\theta(r))^2}{r} \\
& - 10/3 \frac{F_2(r) (\cos(\beta))^2 (a_\theta(r))^2}{r} + 16/3\, P_1(r) (\sin(\beta))^4 (a_z(r))^3 \frac{d}{dr} a_z(r) \\
& - 2/3\, F_1(r) \frac{d}{dr} I_4(r) - \frac{\mu\, (a_\theta(r))^2}{r} - 4/3 \frac{P_1(r) (\cos(\beta))^4 (a_\theta(r))^4}{r} \\
& + 4/3 \frac{P_1(r) (\sin(\beta))^4 (a_z(r))^4}{r} + 4\, \mu\, a_r(r) \frac{d}{dr} a_r(r) \\
& + 4/3 \left(\frac{d}{dr} P_1(r)\right) (\sin(\beta))^4 (a_z(r))^4 + 2 \left(\frac{d}{dr} F_1(r)\right) (\sin(\beta))^2 (a_z(r))^2 \\
& + 4\, F_1(r) (\sin(\beta))^2 a_z(r) \frac{d}{dr} a_z(r) - 2/3 \left(\frac{d}{dr} F_2(r)\right) I_6(r) \\
& - 2/3 \left(\frac{d}{dr} F_1(r)\right) I_4(r) - 2/3\, F_2(r) \frac{d}{dr} I_6(r)
\end{aligned}
$$

$$C_2 = \mu\,(a_r(r))^2 + 4/3\,P_2(r)(\sin(\beta))^2(a_z(r))^2(\cos(\beta))^2(a_\theta(r))^2 + 4/3\,P_1(r)(\sin(\beta))^2(a_z(r))^2(\cos(\beta))^2(a_\theta(r))^2 + 4/3\,P_1(r)(\sin(\beta))^4(a_z(r))^4 + 4/3\,F_1(r)(\sin(\beta))^2(a_z(r))^2 + 4/3\,F_2(r)(\sin(\beta))^2(a_z(r))^2 + 4/3\,P_2(r)(\sin(\beta))^4(a_z(r))^4$$

$$D_2 = -\mu$$

$$a_1 = -4/3\,\frac{F_1(r_{in})(\cos(\beta))^2(a_\theta(r_{in}))^2}{r_{in}} + 4/3\,\frac{P_2(r_{in})(\sin(\beta))^4(a_z(r_{in}))^4}{r_{in}} - 4/3\,\frac{F_2(r_{in})(\cos(\beta))^2(a_\theta(r_{in}))^2}{r_{in}} + 4/3\,\frac{F_1(r_{in})(\sin(\beta))^2(a_z(rin))^2}{r_{in}} - 4/3\,\frac{P_1(rin)(\cos(\beta))^4(a_\theta(r_{in}))^4}{r_{in}} + 4/3\,\frac{P_1(r_{in})(\sin(\beta))^4(a_z(r_{in}))^4}{r_{in}} + 4/3\,\frac{F_2(r_{in})(\sin(\beta))^2(a_z(r_{in}))^2}{r_{in}} - 4/3\,\frac{P_2(r_{in})(\cos(\beta))^4(a_\theta(r_{in}))^4}{r_{in}}$$

$$a_2 = 4/3\,P_1(r_{in})(\sin(\beta))^2(a_z(r_{in}))^2(\cos(\beta))^2(a_\theta(r_{in}))^2 + 4/3\,P_2(rin)(\sin(\beta))^4(a_z(r_{in}))^4 + 4/3\,P_2(r_{in})(\sin(\beta))^2(a_z(r_{in}))^2(\cos(\beta))^2(a_\theta(r_{in}))^2 + 2\,F_1(r_{in})(\sin(\beta))^2(a_z(rin))^2 + 2\,\mu\,q(r_{in}) + 2/3\,F_1(r_{in})(\cos(\beta))^2(a_\theta(r_{in}))^2 + 2\,F_2(r_{in})(\sin(\beta))^2(a_z(r_{in}))^2 + 4/3\,P_1(r_{in})(\sin(\beta))^4(a_z(r_{in}))^4 + 2/3\,F_2(r_{in})(\cos(\beta))^2(a_\theta(r_{in}))^2 + 2/3\,F_1(r_{in})\,I_4(r_{in}) + 2/3\,F_2(r_{in})\,I_6(r_{in})$$

$$a_4 = -\mu$$

$$b_1 = -2\,g\mu\,q(r_{in}) + \frac{\mu\,(a_r(r_{in}))^2}{g{r_{in}}^2} - 2/3\,F_1(r_{in})\,g(\cos(\beta))^2(a_\theta(r_{in}))^2 - 2/3\,F_1(r_{in})\,g(\sin(\beta))^2(a_z(r_{in}))^2 - 2/3\,F_2(r_{in})\,g(\cos(\beta))^2(a_\theta(r_{in}))^2 - 2/3\,F_2(r_{in})\,g(\sin(\beta))^2(a_z(r_{in}))^2 + g\mu\,(a_r(r_{in}))^2 - 2/3\,gF_1(r_{in})\,I_4(r_{in}) - 2/3\,gF_2(r_{in})\,I_6(r_{in})$$

$$b_2 = -\frac{\mu\,(a_r(r_{in}))^2}{gr_{in}}$$

$$b_3 = -\frac{\mu\,(a_r(r_{in}))^2}{g}$$

$$c_5 = -4/3\,\frac{P_2(r_{out})(\cos(\beta))^4(a_\theta(r_{out}))^4}{r_{out}} - 4/3\,\frac{F_1(r_{out})(\cos(\beta))^2(a_\theta(r_{out}))^2}{r_{out}} + 4/3\,\frac{F_1(r_{out})(\sin(\beta))^2(a_z(r_{out}))^2}{r_{out}} + 4/3\,\frac{P_2(r_{out})(\sin(\beta))^4(a_z(r_{out}))^4}{r_{out}} - 4/3\,\frac{F_2(r_{out})(\cos(\beta))^2(a_\theta(r_{out}))^2}{r_{out}} + 4/3\,\frac{F_2(r_{out})(\sin(\beta))^2(a_z(r_{out}))^2}{r_{out}} - 4/3\,\frac{P_1(r_{out})(\cos(\beta))^4(a_\theta(r_{out}))^4}{r_{out}} + 4/3\,\frac{P_1(r_{out})(\sin(\beta))^4(a_z(r_{out}))^4}{r_{out}}$$

$$
\begin{aligned}
c_6 = {} & 4/3\, P_1\left(r_{out}\right)\left(\sin\left(\beta\right)\right)^2\left(a_z\left(r_{out}\right)\right)^2\left(\cos\left(\beta\right)\right)^2\left(a_\theta\left(r_{out}\right)\right)^2 + 4/3\, P_2\left(r_{out}\right)\left(\sin\left(\beta\right)\right)^4\left(a_z\left(r_{out}\right)\right)^4 \\
& + 4/3\, P_2\left(r_{out}\right)\left(\sin\left(\beta\right)\right)^2\left(a_z\left(r_{out}\right)\right)^2\left(\cos\left(\beta\right)\right)^2\left(a_\theta\left(r_{out}\right)\right)^2 + 2\, F_1\left(r_{out}\right)\left(\sin\left(\beta\right)\right)^2\left(a_z\left(r_{out}\right)\right)^2 \\
& + \mu\, q\left(r_{out}\right) + 2/3\, F_1\left(r_{out}\right)\left(\cos\left(\beta\right)\right)^2\left(a_\theta\left(r_{out}\right)\right)^2 + \mu\,\left(a_r\left(r_{out}\right)\right)^2 \\
& + 2\, F_2\left(r_{out}\right)\left(\sin\left(\beta\right)\right)^2\left(a_z\left(r_{out}\right)\right)^2 + 4/3\, P_1\left(r_{out}\right)\left(\sin\left(\beta\right)\right)^4\left(a_z\left(r_{out}\right)\right)^4 \\
& + 2/3\, F_2\left(r_{out}\right)\left(\cos\left(\beta\right)\right)^2\left(a_\theta\left(r_{out}\right)\right)^2 - P_{ext}
\end{aligned}
$$

$$c_8 = -\mu$$

$$
\begin{aligned}
d_5 = {} & -\mu\, q\left(r_{out}\right) g + \frac{\mu\,\left(a_r\left(r_{out}\right)\right)^2}{g{r_{out}}^2} - 2/3\, F_1\left(r_{out}\right) g \left(\cos\left(\beta\right)\right)^2\left(a_\theta\left(r_{out}\right)\right)^2 \\
& - 2/3\, F_1\left(r_{out}\right) g \left(\sin\left(\beta\right)\right)^2\left(a_z\left(r_{out}\right)\right)^2 - 2/3\, F_2\left(r_{out}\right) g \left(\cos\left(\beta\right)\right)^2\left(a_\theta\left(r_{out}\right)\right)^2 \\
& - 2/3\, F_2\left(r_{out}\right) g \left(\sin\left(\beta\right)\right)^2\left(a_z\left(r_{out}\right)\right)^2 + P_{ext}\, g
\end{aligned}
$$

$$d_6 = -\frac{\mu\,\left(a_r\left(r_{out}\right)\right)^2}{g r_{out}}$$

$$d_7 = -\frac{\mu\,\left(a_r\left(r_{out}\right)\right)^2}{g}$$

$$
\begin{aligned}
s_{ur1}(r) = {} & -4/3\, \frac{P_2\left(r\right)\left(\cos\left(\beta\right)\right)^4\left(a_\theta\left(r\right)\right)^4}{r} + 4/3\, \frac{P_1\left(r\right)\left(\sin\left(\beta\right)\right)^4\left(a_z\left(r\right)\right)^4}{r} \\
& - 4/3\, \frac{F_1\left(r\right)\left(\cos\left(\beta\right)\right)^2\left(a_\theta\left(r\right)\right)^2}{r} + 4/3\, \frac{F_1\left(r\right)\left(\sin\left(\beta\right)\right)^2\left(a_z\left(r\right)\right)^2}{r} \\
& + 4/3\, \frac{P_2\left(r\right)\left(\sin\left(\beta\right)\right)^4\left(a_z\left(r\right)\right)^4}{r} - 4/3\, \frac{F_2\left(r\right)\left(\cos\left(\beta\right)\right)^2\left(a_\theta\left(r\right)\right)^2}{r} \\
& + 4/3\, \frac{F_2\left(r\right)\left(\sin\left(\beta\right)\right)^2\left(a_z\left(r\right)\right)^2}{r} - 4/3\, \frac{P_1\left(r\right)\left(\cos\left(\beta\right)\right)^4\left(a_\theta\left(r\right)\right)^4}{r}
\end{aligned}
$$

$$
\begin{aligned}
s_{ur2}(r) = {} & 4/3\, P_1\left(r\right)\left(\sin\left(\beta\right)\right)^2\left(a_z\left(r\right)\right)^2\left(\cos\left(\beta\right)\right)^2\left(a_\theta\left(r\right)\right)^2 \\
& + 4/3\, P_2\left(r\right)\left(\sin\left(\beta\right)\right)^4\left(a_z\left(r\right)\right)^4 \\
& + 4/3\, P_2\left(r\right)\left(\sin\left(\beta\right)\right)^2\left(a_z\left(r\right)\right)^2\left(\cos\left(\beta\right)\right)^2\left(a_\theta\left(r\right)\right)^2 \\
& + 2\, F_1\left(r\right)\left(\sin\left(\beta\right)\right)^2\left(a_z\left(r\right)\right)^2 + \mu\, q\left(r\right) + 2/3\, F_1\left(r\right)\left(\cos\left(\beta\right)\right)^2\left(a_\theta\left(r\right)\right)^2 \\
& + \mu\,\left(a_r\left(r\right)\right)^2 + 2\, F_2\left(r\right)\left(\sin\left(\beta\right)\right)^2\left(a_z\left(r\right)\right)^2 \\
& + 4/3\, P_1\left(r\right)\left(\sin\left(\beta\right)\right)^4\left(a_z\left(r\right)\right)^4 + 2/3\, F_2\left(r\right)\left(\cos\left(\beta\right)\right)^2\left(a_\theta\left(r\right)\right)^2
\end{aligned}
$$

$$
\begin{aligned}
s_{uz1}(r) = {} & -\mu\, q\left(r\right) g + \frac{\mu\,\left(a_r\left(r\right)\right)^2}{g r^2} - 2/3\, F_1\left(r\right) g \left(\cos\left(\beta\right)\right)^2\left(a_\theta\left(r\right)\right)^2 \\
& - 2/3\, F_1\left(r\right) g \left(\sin\left(\beta\right)\right)^2\left(a_z\left(r\right)\right)^2 - 2/3\, F_2\left(r\right) g \left(\cos\left(\beta\right)\right)^2\left(a_\theta\left(r\right)\right)^2 \\
& - 2/3\, F_2\left(r\right) g \left(\sin\left(\beta\right)\right)^2\left(a_z\left(r\right)\right)^2
\end{aligned}
$$

$$s_{uz2}(r) = -\frac{\mu\,\left(a_r\left(r\right)\right)^2}{g r}$$

$$s_{uz3}(r) = -\frac{\mu\,(a_r(r))^2}{g}$$

$$\begin{aligned}
e_1 = &-4/3\,\frac{P_2(r_c)(\cos(\beta))^4(a_\theta(r_c))^4}{r_c} + 4/3\,\frac{P_1(r_c)(\sin(\beta))^4(a_z(r_c))^4}{r_c}\\
&- 4/3\,\frac{F_1(r_c)(\cos(\beta))^2(a_\theta(r_c))^2}{r_c} + 4/3\,\frac{F_1(r_c)(\sin(\beta))^2(a_z(r_c))^2}{r_c}\\
&+ 4/3\,\frac{P_2(r_c)(\sin(\beta))^4(a_z(r_c))^4}{r_c} - 4/3\,\frac{F_2(r_c)(\cos(\beta))^2(a_\theta(r_c))^2}{r_c}\\
&+ 4/3\,\frac{F_2(r_c)(\sin(\beta))^2(a_z(r_c))^2}{r_c} - 4/3\,\frac{P_1(r_c)(\cos(\beta))^4(a_\theta(r_c))^4}{r_c}
\end{aligned}$$

$$\begin{aligned}
e_2 = &\,4/3\,P_1(r_c)(\sin(\beta))^2(a_z(r_c))^2(\cos(\beta))^2(a_\theta(r_c))^2 + 4/3\,P_2(r_c)(\sin(\beta))^4(a_z(r_c))^4\\
&+ 4/3\,P_2(r_c)(\sin(\beta))^2(a_z(r_c))^2(\cos(\beta))^2(a_\theta(r_c))^2 + 2\,F_1(r_c)(\sin(\beta))^2(a_z(r_c))^2 + \mu Y_{b9}\\
&+ 2/3\,F_1(r_c)(\cos(\beta))^2(a_\theta(r_c))^2 + \mu\,(a_r(r_c))^2 + 2\,F_2(r_c)(\sin(\beta))^2(a_z(r_c))^2\\
&+ 4/3\,P_1(r_c)(\sin(\beta))^4(a_z(r_c))^4 + 2/3\,F_2(r_c)(\cos(\beta))^2(a_\theta(r_c))^2
\end{aligned}$$

$$e_4 = \mu$$

$$\begin{aligned}
e_5 = &-4/3\,\frac{P_2(r_c)(\cos(\beta))^4(a_\theta(r_c))^4}{r_c} + 4/3\,\frac{P_1(r_c)(\sin(\beta))^4(a_z(r_c))^4}{r_c}\\
&- 4/3\,\frac{F_1(r_c)(\cos(\beta))^2(a_\theta(r_c))^2}{r_c} + 4/3\,\frac{F_1(r_c)(\sin(\beta))^2(a_z(r_c))^2}{r_c}\\
&+ 4/3\,\frac{P_2(r_c)(\sin(\beta))^4(a_z(r_c))^4}{r_c} - 4/3\,\frac{F_2(r_c)(\cos(\beta))^2(a_\theta(r_c))^2}{r_c}\\
&+ 4/3\,\frac{F_2(r_c)(\sin(\beta))^2(a_z(r_c))^2}{r_c} - 4/3\,\frac{P_1(r_c)(\cos(\beta))^4(a_\theta(r_c))^4}{r_c}
\end{aligned}$$

$$\begin{aligned}
e_6 = &\,4/3\,P_1(r_c)(\sin(\beta))^2(a_z(r_c))^2(\cos(\beta))^2(a_\theta(r_c))^2 + 4/3\,P_2(r_c)(\sin(\beta))^4(a_z(r_c))^4\\
&+ 4/3\,P_2(r_c)(\sin(\beta))^2(a_z(r_c))^2(\cos(\beta))^2(a_\theta(r_c))^2 + 2\,F_1(r_c)(\sin(\beta))^2(a_z(r_c))^2 + \mu Y_{b10}\\
&+ 2/3\,F_1(r_c)(\cos(\beta))^2(a_\theta(r_c))^2 + \mu\,(a_r(r_c))^2 + 2\,F_2(r_c)(\sin(\beta))^2(a_z(r_c))^2\\
&+ 4/3\,P_1(r_c)(\sin(\beta))^4(a_z(r_c))^4 + 2/3\,F_2(r_c)(\cos(\beta))^2(a_\theta(r_c))^2
\end{aligned}$$

$$e_8 = \mu$$

$$\begin{aligned}
f_1 = &-\mu\,Y_{b9}g + \frac{\mu\,(a_r(r_c))^2}{g{r_c}^2} - 2/3\,F_1(r_c)\,g\,(\cos(\beta))^2(a_\theta(r_c))^2\\
&- 2/3\,F_1(r_c)\,g\,(\sin(\beta))^2(a_z(r_c))^2 - 2/3\,F_2(r_c)\,g\,(\cos(\beta))^2(a_\theta(r_c))^2\\
&- 2/3\,F_2(r_c)\,g\,(\sin(\beta))^2(a_z(r_c))^2
\end{aligned}$$

$$f_2 = -\frac{\mu\,(a_r(r_c))^2}{g r_c}$$

$$f_3 = -\frac{\mu\,(a_r\,(r_c))^2}{g}$$

$$f_5 = -\mu\,Y_{b10}g + \frac{\mu\,(a_r\,(r_c))^2}{g{r_c}^2} - 2/3\,F_1\,(r_c)\,g\,(\cos(\beta))^2\,(a_\theta\,(r_c))^2 \\ - 2/3\,F_1\,(r_c)\,g\,(\sin(\beta))^2\,(a_z\,(r_c))^2 - 2/3\,F_2\,(r_c)\,g\,(\cos(\beta))^2\,(a_\theta\,(r_c))^2 \\ - 2/3\,F_2\,(r_c)\,g\,(\sin(\beta))^2\,(a_z\,(r_c))^2$$

$$f_6 = -\frac{\mu\,(a_r\,(r_c))^2}{g r_c}$$

$$f_7 = -\frac{\mu\,(a_r\,(r_c))^2}{g}$$

3 Coefficients for incremental inner pressure

The coefficients for incremental inner pressure are the parameters in Section 6.

$$f_5(r) = 2\,a_r\,(r)\,\frac{d}{dr}a_r\,(r) - 2/3\,\frac{\left(\frac{d}{dr}F_1\,(r)\right)I_4\,(r)}{\mu} - 2/3\,\frac{F_1\,(r)\,\frac{d}{dr}I_4\,(r)}{\mu} \\ - 2/3\,\frac{\left(\frac{d}{dr}F_2\,(r)\right)I_6\,(r)}{\mu} - 2/3\,\frac{F_2\,(r)\,\frac{d}{dr}I_6\,(r)}{\mu} + \frac{(a_r\,(r))^2}{r} \\ - \frac{(a_\theta\,(r))^2}{r} - 2\,\frac{F_1\,(r)\,(\cos(\beta))^2\,(a_\theta\,(r))^2}{\mu\,r} \\ - 2\,\frac{F_2\,(r)\,(\cos(\beta))^2\,(a_\theta\,(r))^2}{\mu\,r}$$

$$
\begin{aligned}
f_2(r) = & -4/3\,\frac{P_1(r)(\cos(\beta))^4(a_\theta(r))^4}{\mu\,r^2} - 4/3\,\frac{P_2(r)(\cos(\beta))^4(a_\theta(r))^4}{\mu\,r^2} \\
& + 8/3\,\frac{P_1(r)(\cos(\beta))^2(a_\theta(r))^2(\sin(\beta))^2(a_z(r))^2}{\mu\,r^2} \\
& + 8/3\,\frac{P_2(r)(\cos(\beta))^2(a_\theta(r))^2(\sin(\beta))^2(a_z(r))^2}{\mu\,r^2} \\
& - 4\,\frac{F_2(r)(\cos(\beta))^2 a_\theta(r)\frac{d}{dr}a_\theta(r)}{\mu\,r} - 4/3\,\frac{F_2(r)(\sin(\beta))^2 a_z(r)\frac{d}{dr}a_z(r)}{\mu\,r} \\
& - 4/3\,\frac{\left(\frac{d}{dr}P_1(r)\right)(\cos(\beta))^2(a_\theta(r))^2(\sin(\beta))^2(a_z(r))^2}{\mu\,r} \\
& - 16/3\,\frac{P_1(r)(\cos(\beta))^4(a_\theta(r))^3\frac{d}{dr}a_\theta(r)}{\mu\,r} \\
& - 8/3\,\frac{P_1(r)(\cos(\beta))^2 a_\theta(r)\left(\frac{d}{dr}a_\theta(r)\right)(\sin(\beta))^2(a_z(r))^2}{\mu\,r} \\
& - 8/3\,\frac{P_1(r)(\cos(\beta))^2(a_\theta(r))^2(\sin(\beta))^2 a_z(r)\frac{d}{dr}a_z(r)}{\mu\,r} \\
& - 4/3\,\frac{\left(\frac{d}{dr}P_2(r)\right)(\cos(\beta))^2(a_\theta(r))^2(\sin(\beta))^2(a_z(r))^2}{\mu\,r} \\
& - 16/3\,\frac{P_2(r)(\cos(\beta))^4(a_\theta(r))^3\frac{d}{dr}a_\theta(r)}{\mu\,r} \\
& - 4/3\,\frac{\left(\frac{d}{dr}P_1(r)\right)(\cos(\beta))^4(a_\theta(r))^4}{\mu\,r} - 4/3\,\frac{\left(\frac{d}{dr}P_2(r)\right)(\cos(\beta))^4(a_\theta(r))^4}{\mu\,r} \\
& - 2\,\frac{\left(\frac{d}{dr}F_1(r)\right)(\cos(\beta))^2(a_\theta(r))^2}{\mu\,r} - 2/3\,\frac{\left(\frac{d}{dr}F_1(r)\right)(\sin(\beta))^2(a_z(r))^2}{\mu\,r} \\
& - 2\,\frac{\left(\frac{d}{dr}F_2(r)\right)(\cos(\beta))^2(a_\theta(r))^2}{\mu\,r} - 2/3\,\frac{\left(\frac{d}{dr}F_2(r)\right)(\sin(\beta))^2(a_z(r))^2}{\mu\,r} \\
& + 8/3\,\frac{F_1(r)(\cos(\beta))^2(a_\theta(r))^2}{\mu\,r^2} + 8/3\,\frac{F_2(r)(\cos(\beta))^2(a_\theta(r))^2}{\mu\,r^2} + 2/3\,\frac{\left(\frac{d}{dr}F_1(r)\right)I_4(r)}{\mu\,r} \\
& + 2/3\,\frac{F_1(r)\frac{d}{dr}I_4(r)}{\mu\,r} + 2/3\,\frac{\left(\frac{d}{dr}F_2(r)\right)I_6(r)}{\mu\,r} + 2/3\,\frac{F_2(r)\frac{d}{dr}I_6(r)}{\mu\,r} \\
& - 8/3\,\frac{P_2(r)(\cos(\beta))^2 a_\theta(r)\left(\frac{d}{dr}a_\theta(r)\right)(\sin(\beta))^2(a_z(r))^2}{\mu\,r} \\
& - 8/3\,\frac{P_2(r)(\cos(\beta))^2(a_\theta(r))^2(\sin(\beta))^2 a_z(r)\frac{d}{dr}a_z(r)}{\mu\,r} - 4\,\frac{F_1(r)(\cos(\beta))^2 a_\theta(r)\frac{d}{dr}a_\theta(r)}{\mu\,r} \\
& - 4/3\,\frac{F_1(r)(\sin(\beta))^2 a_z(r)\frac{d}{dr}a_z(r)}{\mu\,r} - 4\,\frac{a_r(r)\frac{d}{dr}a_r(r)}{r}
\end{aligned}
$$

$$
\begin{aligned}
p_1(r_{in}) = & -2\,\frac{\mu\,Y_{a5}}{r_{in}} - 2/3\,\frac{F_1\left(r_{in}\right)\left(\sin\left(\beta\right)\right)^2\left(a_z\left(r_{in}\right)\right)^2}{r_{in}} \\
& - 2/3\,\frac{F_2\left(r_{in}\right)\left(\sin\left(\beta\right)\right)^2\left(a_z\left(r_{in}\right)\right)^2}{r_{in}} - 2\,\frac{F_2\left(r_{in}\right)\left(\cos\left(\beta\right)\right)^2\left(a_\theta\left(r_{in}\right)\right)^2}{r_{in}} \\
& - 4/3\,\frac{P_1\left(r_{in}\right)\left(\cos\left(\beta\right)\right)^4\left(a_\theta\left(r_{in}\right)\right)^4}{r_{in}} - 2\,\frac{F_1\left(r_{in}\right)\left(\cos\left(\beta\right)\right)^2\left(a_\theta\left(r_{in}\right)\right)^2}{r_{in}} \\
& - 4/3\,\frac{P_2\left(r_{in}\right)\left(\cos\left(\beta\right)\right)^4\left(a_\theta\left(r_{in}\right)\right)^4}{r_{in}} \\
& - 4/3\,\frac{P_2\left(r_{in}\right)\left(\cos\left(\beta\right)\right)^2\left(a_\theta\left(r_{in}\right)\right)^2\left(\sin\left(\beta\right)\right)^2\left(a_z\left(r_{in}\right)\right)^2}{r_{in}} \\
& - 2/3\,\frac{F_1\left(r_{in}\right)I_4\left(r_{in}\right)}{r_{in}} - 2/3\,\frac{F_2\left(r_{in}\right)I_6\left(r_{in}\right)}{r_{in}} \\
& - 4/3\,\frac{P_1\left(r_{in}\right)\left(\cos\left(\beta\right)\right)^2\left(a_\theta\left(r_{in}\right)\right)^2\left(\sin\left(\beta\right)\right)^2\left(a_z\left(r_{in}\right)\right)^2}{r_{in}}
\end{aligned}
$$

$$
\begin{aligned}
p_2(r_{in}) = & -\frac{\mu\,Y_{a6}}{r_{out}} - \frac{\mu\,\left(a_r\left(r_{out}\right)\right)^2}{r_{out}} - 4/3\,\frac{P_1\left(r_{out}\right)\left(\cos\left(\beta\right)\right)^4\left(a_\theta\left(r_{out}\right)\right)^4}{r_{out}} \\
& - 4/3\,\frac{P_1\left(r_{out}\right)\left(\cos\left(\beta\right)\right)^2\left(a_\theta\left(r_{out}\right)\right)^2\left(\sin\left(\beta\right)\right)^2\left(a_z\left(r_{out}\right)\right)^2}{r_{out}} \\
& - 4/3\,\frac{P_2\left(r_{out}\right)\left(\cos\left(\beta\right)\right)^4\left(a_\theta\left(r_{out}\right)\right)^4}{r_{out}} \\
& - 4/3\,\frac{P_2\left(r_{out}\right)\left(\cos\left(\beta\right)\right)^2\left(a_\theta\left(r_{out}\right)\right)^2\left(\sin\left(\beta\right)\right)^2\left(a_z\left(r_{out}\right)\right)^2}{r_{out}} \\
& - 2\,\frac{F_1\left(r_{out}\right)\left(\cos\left(\beta\right)\right)^2\left(a_\theta\left(r_{out}\right)\right)^2}{r_{out}} - 2/3\,\frac{F_1\left(r_{out}\right)\left(\sin\left(\beta\right)\right)^2\left(a_z\left(r_{out}\right)\right)^2}{r_{out}} \\
& - 2\,\frac{F_2\left(r_{out}\right)\left(\cos\left(\beta\right)\right)^2\left(a_\theta\left(r_{out}\right)\right)^2}{r_{out}} - 2/3\,\frac{F_2\left(r_{out}\right)\left(\sin\left(\beta\right)\right)^2\left(a_z\left(r_{out}\right)\right)^2}{r_{out}} + \frac{P_{ext}}{r_{out}}
\end{aligned}
$$

$$
\begin{aligned}
p_3(r_c) = & -\frac{\mu\,Y_{b5}}{r_c} - \frac{\mu\,\left(a_r\left(r_c\right)\right)^2}{r_c} - 4/3\,\frac{P_1\left(r_c\right)\left(\cos\left(\beta\right)\right)^4\left(a_\theta\left(r_c\right)\right)^4}{r_c} \\
& - 4/3\,\frac{P_1\left(r_c\right)\left(\cos\left(\beta\right)\right)^2\left(a_\theta\left(r_c\right)\right)^2\left(\sin\left(\beta\right)\right)^2\left(a_z\left(r_c\right)\right)^2}{r_c} - 4/3\,\frac{P_2\left(r_c\right)\left(\cos\left(\beta\right)\right)^4\left(a_\theta\left(r_c\right)\right)^4}{r_c} \\
& - 4/3\,\frac{P_2\left(r_c\right)\left(\cos\left(\beta\right)\right)^2\left(a_\theta\left(r_c\right)\right)^2\left(\sin\left(\beta\right)\right)^2\left(a_z\left(r_c\right)\right)^2}{r_c} - 2\,\frac{F_1\left(r_c\right)\left(\cos\left(\beta\right)\right)^2\left(a_\theta\left(r_c\right)\right)^2}{r_c} \\
& - 2/3\,\frac{F_1\left(r_c\right)\left(\sin\left(\beta\right)\right)^2\left(a_z\left(r_c\right)\right)^2}{r_c} - 2\,\frac{F_2\left(r_c\right)\left(\cos\left(\beta\right)\right)^2\left(a_\theta\left(r_c\right)\right)^2}{r_c} \\
& - 2/3\,\frac{F_2\left(r_c\right)\left(\sin\left(\beta\right)\right)^2\left(a_z\left(r_c\right)\right)^2}{r_c}
\end{aligned}
$$

$$
\begin{aligned}
p_4(r_c) = &-\frac{\mu Y_{b6}}{r_c} - \frac{\mu \left(a_r\left(r_c\right)\right)^2}{r_c} - 4/3\,\frac{P_1\left(r_c\right)\left(\cos\left(\beta\right)\right)^4\left(a_\theta\left(r_c\right)\right)^4}{r_c}\\
&- 4/3\,\frac{P_1\left(r_c\right)\left(\cos\left(\beta\right)\right)^2\left(a_\theta\left(r_c\right)\right)^2\left(\sin\left(\beta\right)\right)^2\left(a_z\left(r_c\right)\right)^2}{r_c}\\
&- 4/3\,\frac{P_2\left(r_c\right)\left(\cos\left(\beta\right)\right)^4\left(a_\theta\left(r_c\right)\right)^4}{r_c}\\
&- 4/3\,\frac{P_2\left(r_c\right)\left(\cos\left(\beta\right)\right)^2\left(a_\theta\left(r_c\right)\right)^2\left(\sin\left(\beta\right)\right)^2\left(a_z\left(r_c\right)\right)^2}{r_c} - 2\,\frac{F_1\left(r_c\right)\left(\cos\left(\beta\right)\right)^2\left(a_\theta\left(r_c\right)\right)^2}{r_c}\\
&- 2/3\,\frac{F_1\left(r_c\right)\left(\sin\left(\beta\right)\right)^2\left(a_z\left(r_c\right)\right)^2}{r_c} - 2\,\frac{F_2\left(r_c\right)\left(\cos\left(\beta\right)\right)^2\left(a_\theta\left(r_c\right)\right)^2}{r_c}\\
&- 2/3\,\frac{F_2\left(r_c\right)\left(\sin\left(\beta\right)\right)^2\left(a_z\left(r_c\right)\right)^2}{r_c}
\end{aligned}
$$

$$
\begin{aligned}
S_1(r) = &-\frac{\mu\, q\left(r\right)}{r} - \frac{\mu \left(a_r\left(r\right)\right)^2}{r} - 4/3\,\frac{P_1\left(r\right)\left(\cos\left(\beta\right)\right)^4\left(a_\theta\left(r\right)\right)^4}{r}\\
&- 4/3\,\frac{P_1\left(r\right)\left(\cos\left(\beta\right)\right)^2\left(a_\theta\left(r\right)\right)^2\left(\sin\left(\beta\right)\right)^2\left(a_z\left(r\right)\right)^2}{r}\\
&- 4/3\,\frac{P_2\left(r\right)\left(\cos\left(\beta\right)\right)^4\left(a_\theta\left(r\right)\right)^4}{r}\\
&- 4/3\,\frac{P_2\left(r\right)\left(\cos\left(\beta\right)\right)^2\left(a_\theta\left(r\right)\right)^2\left(\sin\left(\beta\right)\right)^2\left(a_z\left(r\right)\right)^2}{r}\\
&- 2\,\frac{F_1\left(r\right)\left(\cos\left(\beta\right)\right)^2\left(a_\theta\left(r\right)\right)^2}{r} - 2/3\,\frac{F_1\left(r\right)\left(\sin\left(\beta\right)\right)^2\left(a_z\left(r\right)\right)^2}{r}\\
&- 2\,\frac{F_2\left(r\right)\left(\cos\left(\beta\right)\right)^2\left(a_\theta\left(r\right)\right)^2}{r} - 2/3\,\frac{F_2\left(r\right)\left(\sin\left(\beta\right)\right)^2\left(a_z\left(r\right)\right)^2}{r}
\end{aligned}
$$

$$
\begin{aligned}
A(r) = &\,2\,a_r\left(r\right)\frac{d}{dr}a_r\left(r\right) - 2/3\,\frac{\left(\frac{d}{dr}F_1\left(r\right)\right)I_4\left(r\right)}{\mu} - 2/3\,\frac{F_1\left(r\right)\frac{d}{dr}I_4\left(r\right)}{\mu}\\
&- 2/3\,\frac{\left(\frac{d}{dr}F_2\left(r\right)\right)I_6\left(r\right)}{\mu} - 2/3\,\frac{F_2\left(r\right)\frac{d}{dr}I_6\left(r1\right)}{\mu} + \frac{\left(a_r\left(r\right)\right)^2}{r}\\
&- \frac{\left(a_\theta\left(r\right)\right)^2}{r} - 2\,\frac{F_1\left(r\right)\left(\cos\left(\beta\right)\right)^2\left(a_\theta\left(r\right)\right)^2}{\mu\, r} - 2\,\frac{F_2\left(r\right)\left(\cos\left(\beta\right)\right)^2\left(a_\theta\left(r\right)\right)^2}{\mu\, r}
\end{aligned}
$$

$$
\begin{aligned}
B(r) = {} & -8/3\,\frac{P_1(r)(\cos(\beta))^2 a_\theta(r)\left(\frac{d}{dr}a_\theta(r)\right)(\sin(\beta))^2 (a_z(r))^2}{r} \\
& + 8/3\,\frac{P_2(r)(\cos(\beta))^2 (a_\theta(r))^2 (\sin(\beta))^2 (a_z(r))^2}{r^2} - 16/3\,\frac{P_1(r)(\cos(\beta))^4 (a_\theta(r))^3 \frac{d}{dr}a_\theta(r)}{r} \\
& - 4/3\,\frac{\left(\frac{d}{dr}P_1(r)\right)(\cos(\beta))^2 (a_\theta(r))^2 (\sin(\beta))^2 (a_z(r))^2}{r} \\
& - 16/3\,\frac{P_2(r)(\cos(\beta))^4 (a_\theta(r))^3 \frac{d}{dr}a_\theta(r)}{r} \\
& - 8/3\,\frac{P_2(r)(\cos(\beta))^2 a_\theta(r)\left(\frac{d}{dr}a_\theta(r)\right)(\sin(\beta))^2 (a_z(r))^2}{r} \\
& - 8/3\,\frac{P_2(r)(\cos(\beta))^2 (a_\theta(r))^2 (\sin(\beta))^2 a_z(r)\frac{d}{dr}a_z(r)}{r} \\
& + 8/3\,\frac{P_1(r)(\cos(\beta))^2 (a_\theta(r))^2 (\sin(\beta))^2 (a_z(r))^2}{r^2} \\
& - 8/3\,\frac{P_1(r)(\cos(\beta))^2 (a_\theta(r))^2 (\sin(\beta))^2 a_z(r)\frac{d}{dr}a_z(r)}{r} \\
& - 4/3\,\frac{\left(\frac{d}{dr}P_2(r)\right)(\cos(\beta))^2 (a_\theta(r))^2 (\sin(\beta))^2 (a_z(r))^2}{r} \\
& - 4\,\frac{F_2(r)(\cos(\beta))^2 a_\theta(r)\frac{d}{dr}a_\theta(r)}{r} - 4/3\,\frac{F_2(r)(\sin(\beta))^2 a_z(r)\frac{d}{dr}a_z(r)}{r} \\
& - 4\,\frac{F_1(r)(\cos(\beta))^2 a_\theta(r)\frac{d}{dr}a_\theta(r)}{r} - 4/3\,\frac{F_1(r)(\sin(\beta))^2 a_z(r)\frac{d}{dr}a_z(r)}{r} \\
& - 4/3\,\frac{\left(\frac{d}{dr}P_1(r)\right)(\cos(\beta))^4 (a_\theta(r))^4}{r} + 8/3\,\frac{F_1(r)(\cos(\beta))^2 (a_\theta(r))^2}{r^2} - 4\,\frac{\mu\, a_r(r)\frac{d}{dr}a_r(r)}{r} \\
& - 4/3\,\frac{\left(\frac{d}{dr}P_2(r)\right)(\cos(\beta))^4 (a_\theta(r))^4}{r} - 4/3\,\frac{P_2(r)(\cos(\beta))^4 (a_\theta(r))^4}{r^2} \\
& - 2\,\frac{\left(\frac{d}{dr}F_1(r)\right)(\cos(\beta))^2 (a_\theta(r))^2}{r} + 8/3\,\frac{F_2(r)(\cos(\beta))^2 (a_\theta(r))^2}{r^2} \\
& - 4/3\,\frac{P_1(r)(\cos(\beta))^4 (a_\theta(r))^4}{r^2} - 2/3\,\frac{\left(\frac{d}{dr}F_1(r)\right)(\sin(\beta))^2 (a_z(r))^2}{r} \\
& - 2\,\frac{\left(\frac{d}{dr}F_2(r)\right)(\cos(\beta))^2 (a_\theta(r))^2}{r} - 2/3\,\frac{\left(\frac{d}{dr}F_2(r)\right)(\sin(\beta))^2 (a_z(r))^2}{r} + 2/3\,\frac{\left(\frac{d}{dr}F_1(r)\right)I_4(r)}{r} \\
& + 2/3\,\frac{\left(\frac{d}{dr}F_2(r)\right)I_6(r)}{r} + 2/3\,\frac{F_2(r)\frac{d}{dr}I_6(r)}{r} + 2/3\,\frac{F_1(r)\frac{d}{dr}I_4(r)}{r}
\end{aligned}
$$

$$
\begin{aligned}
a(r) = {} & -2\,\frac{\mu\,Y_{a1}}{r_{in}} - 4/3\,\frac{P_1\,(r_{in})\,(\cos(\beta))^4\,(a_\theta\,(r_{in}))^4}{r_{in}} \\
& - 4/3\,\frac{P_1\,(r_{in})\,(\cos(\beta))^2\,(a_\theta\,(r_{in}))^2\,(\sin(\beta))^2\,(a_z\,(r_{in}))^2}{r_{in}} \\
& - 4/3\,\frac{P_2\,(r_{in})\,(\cos(\beta))^4\,(a_\theta\,(r_{in}))^4}{r_{in}} \\
& - 4/3\,\frac{P_2\,(r_{in})\,(\cos(\beta))^2\,(a_\theta\,(r_{in}))^2\,(\sin(\beta))^2\,(a_z\,(r_{in}))^2}{r_{in}} \\
& - 2\,\frac{F_1\,(r_{in})\,(\cos(\beta))^2\,(a_\theta\,(r_{in}))^2}{r_{in}} - 2/3\,\frac{F_1\,(r_{in})\,(\sin(\beta))^2\,(a_z\,(r_{in}))^2}{r_{in}} \\
& - 2\,\frac{F_2\,(r_{in})\,(\cos(\beta))^2\,(a_\theta\,(r_{in}))^2}{r_{in}} - 2/3\,\frac{F_2\,(r_{in})\,(\sin(\beta))^2\,(a_z\,(r_{in}))^2}{r_{in}} \\
& - 2/3\,\frac{F_1\,(r_{in})\,I_4\,(r_{in})}{r_{in}} - 2/3\,\frac{F_2\,(r_{in})\,I_6\,(r_{in})}{r_{in}}
\end{aligned}
$$

$$
\begin{aligned}
b(r) = {} & -\frac{\mu\,Y_{a1}}{r_{out}} - \frac{\mu\,(a_r\,(r_{out}))^2}{r_{out}} - 4/3\,\frac{P_1\,(r_{out})\,(\cos(\beta))^4\,(a_\theta\,(r_{out}))^4}{r_{out}} \\
& - 4/3\,\frac{P_1\,(r_{out})\,(\cos(\beta))^2\,(a_\theta\,(r_{out}))^2\,(\sin(\beta))^2\,(a_z\,(r_{out}))^2}{r_{out}} \\
& - 4/3\,\frac{P_2\,(r_{out})\,(\cos(\beta))^4\,(a_\theta\,(r_{out}))^4}{r_{out}} \\
& - 4/3\,\frac{P_2\,(r_{out})\,(\cos(\beta))^2\,(a_\theta\,(r_{out}))^2\,(\sin(\beta))^2\,(a_z\,(r_{out}))^2}{r_{out}} \\
& - 2\,\frac{F_1\,(r_{out})\,(\cos(\beta))^2\,(a_\theta\,(r_{out}))^2}{r_{out}} - 2/3\,\frac{F_1\,(r_{out})\,(\sin(\beta))^2\,(a_z\,(r_{out}))^2}{r_{out}} \\
& - 2\,\frac{F_2\,(r_{out})\,(\cos(\beta))^2\,(a_\theta\,(r_{out}))^2}{r_{out}} - 2/3\,\frac{F_2\,(r_{out})\,(\sin(\beta))^2\,(a_z\,(r_{out}))^2}{r_{out}} + \frac{P_{ext}}{r_{out}}
\end{aligned}
$$
